# Comprehensive Bird Preservation at Wind Farms

**DOI:** 10.3390/s21010267

**Published:** 2021-01-03

**Authors:** Dawid Gradolewski, Damian Dziak, Milosz Martynow, Damian Kaniecki, Aleksandra Szurlej-Kielanska, Adam Jaworski, Wlodek J. Kulesza

**Affiliations:** 1 Bioseco Sp. z. o. o., Budowlanych 68, 80-298 Gdansk, Poland; damian.dziak@bioseco.com (D.D.); milosz.martynow@bioseco.com (M.M.); damian.kaniecki@bioseco.com (D.K.); adam.jaworski@bioseco.com (A.J.); 2 Department of Mathematics and Natural Sciences, Blekinge Institute of Technology, 371 79 Karlskrona, Sweden; wlodek.kulesza@bth.se; 3 Department of Vertebrate Ecology and Zoology, University of Gdansk, Wita Stwosza 59, 80-308 Gdansk, Poland; tactus@tactus.pl

**Keywords:** artificial intelligence, bird monitoring system, distributed computing, environmental sustainability, monitoring of avifauna, safety system, stereo-vision, vision system

## Abstract

Wind as a clean and renewable energy source has been used by humans for centuries. However, in recent years with the increase in the number and size of wind turbines, their impact on avifauna has become worrisome. Researchers estimated that in the U.S. up to 500,000 birds die annually due to collisions with wind turbines. This article proposes a system for mitigating bird mortality around wind farms. The solution is based on a stereo-vision system embedded in distributed computing and IoT paradigms. After a bird’s detection in a defined zone, the decision-making system activates a collision avoidance routine composed of light and sound deterrents and the turbine stopping procedure. The development process applies a User-Driven Design approach along with the process of component selection and heuristic adjustment. This proposal includes a bird detection method and localization procedure. The bird identification is carried out using artificial intelligence algorithms. Validation tests with a fixed-wing drone and verifying observations by ornithologists proved the system’s desired reliability of detecting a bird with wingspan over 1.5 m from at least 300 m. Moreover, the suitability of the system to classify the size of the detected bird into one of three wingspan categories, small, medium and large, was confirmed.

## 1. Introduction

With the growth of the human population, the robust expansion of urban facilities such as wind farms, power lines and airports into the natural environment of animals, particularly birds and bats, may be observed [1,2,3,4,5,6,7]. Therefore, mutual cohabitation of wildlife and humans increasingly leads to unwanted conflicts and close contact. Bird strikes with synthetic structures are dangerous situations for both. On the one hand, turbine damage and airplane crashes cause human problems. On the other hand, human expansion inflicts itself on the local ecosystem leading not only to habitat loss and fragmentation but above all to the suffering and death of birds [8].

Although we may believe that wind power is a green and renewable energy source, it may also cause the death of rare species of birds and bats. Rotating high speed wind turbine blades are hardly visible for hunting predatory birds. It is hard to estimate the accurate mortality rate, but the most recent studies show that in the U.S. between 140,000 and 500,000 birds die annually [9,10,11]. With the increase of wind energy capacity, this number could even reach 1.4 million. Therefore, there is an immediate need for technical solutions mitigating the impact of wind turbines on local avifauna [9].

Sustainable development requires not only the reduction of carbon dioxide emissions, but it must be conducted without the depletion of nature and wildlife [12]. Therefore, among other things, humans need to develop sustainable, efficient and nature friendly methods and instruments, helping to mitigate the impact of synthetic structures and machines on the whole avifauna.

One of the main technological approaches to achieving Avifauna Individuals Avoidance (AIA) is the Automated Detection and Reaction Method (ADaRM). The ADaRM might be based on the detection of birds and/or bats using machine vision and appropriate reactions to achieve AIA. Such reactions may use light deterrents and/or sound signals, the slowing or stopping of a wind turbine, the delayed takeoff or landing of an aircraft, or the calling of a falconer to scare resting or foraging birds. A solution based on this approach using a stereo-vision system embedded in distributed computing and IoT paradigms is presented in this paper. The system development process applies a User-Driven Design method. The bird detection, localization and identification are carried out using vision methods and artificial intelligence algorithms. Validation tests with a fixed-wing drone and verifying observations by ornithologists proved the system can protect birds around wind farms, with the desired reliability.

## 2. Survey of Related Work

### 2.1. Collision Prevention

There has been several research papers regarding the effective bird protection on wind farms [13,14,15]. So far, the solution preferable by ornithologists is periodic turbine shut-downs during specific weather conditions. The shut-down of turbines is also obligatory during spring and autumn migrations. However, this solution limits the power production of the wind farm and thus the operators’ profits. Therefore, an automatic collision prevention system that could reduce the bird mortality is the subject of research for many scientists and engineers [16].

Pulsing light is one of the methods of repelling birds. Such a solution is widespread in airports to prevent bird collisions with airplanes. Blackwell et al. [17] show that birds register pulsating lights quicker than static lights. Moreover, they claim that the best repelling reaction may be obtained for lights from the wavelength range of 380 nm–400 nm. Doppler et al. [18] as a continuation of the research, applied light at a wavelength of 470 nm, obtaining promising results. In the most recent works Goller et al. [19] tested LED light at 380 nm, 470 nm, 525 nm and 630 nm. It was shown that the best results were obtained applying 470 nm and 630 nm LED light. Moreover, their research showed that waves of 380 nm and 525 nm may actually be luring to birds.

Another tested method of repelling birds has been sound repellents. Bishop et al. [20] show that high frequency sounds and ultrasounds are either inefficient or even dangerous to the birds. They also proved that lower frequencies of sound deter birds more efficiently. However, they observed a habituation effect for birds subjected to longer emissions of the same sound.

Cadets of the Air Force Academy proposed combining pulsing lights with sounds [21]. They obtained good effects in repelling birds using white light, and sound of 2 kHz at a strength of between 90 dB and 135 dB. The presented research shows that it is possible to repel birds from the wind turbine vicinity; however, to reduce the habituation effect it is recommended that the repelling method is only used when a bird is approaching the turbine. Moreover, to ensure enough reaction time for the turbine stopping routine, the bird needs to be detected from a sufficient distance away, which can vary for different species [16].

### 2.2. Detection Methods

The very first automated detection system for birds was created in the 1950s, and was mostly based on radar [22,23]. The interest in the bird detection problem was aroused with the growth in aviation and the subsequent increase in bird strikes. The radar systems can detect any flying object in the monitoring area and estimate the object’s position, velocity and movement [3]. The detection range depends on several factors including the system frequency band, beam angle and power, and antenna size. Presently, bird detection systems allow observations up to 5 km [24]. However, radars are not able to perform direct classification of the species or to distinguish birds from flying objects e.g., drones. Therefore, detailed analysis of the data obtained is still required e.g., through biologist consultation [3]. Moreover, the price, the size of the system, the power consumption, and government emissions regulations limiting the beam frequency and power are the main barrier to wide-scale application of radar for bird detection [25].

Despite the limitations, radar is widely used for bird observations [23,24]. Nevertheless, in the last decade, with the development of image processing algorithms, Artificial Intelligence (AI) and advantages of Graphics Processing Units (GPU) capabilities, vision-based detection systems are becoming more and more powerful [26]. There are two well-known vision detection approaches applied in industrial applications—the single and stereoscopic methods. A single camera unit can detect bird movement and carry out species identification. Such an approach finds use in aerial systems [27,28] and in low budget detection systems [4]. However, the most recent systems use stereoscopy, which extends the single camera system capabilities with additional position and size information for the detected birds [29,30]. Presently, high-resolution cameras coupled in stereoscopic mode may ensure similar distance estimation performance to radar systems [31]. Although the detection range of the vision-based system is limited up to 1.0 km [30]. The main advantage of the vision approach over the radar one, is its ability to detect a single bird or bat, which then can be followed by their identification [32].

In recent years, several approaches have been developed to solve the vision-based bird detection problem on wind farms. Companies such as DT Bird [33], SafeWind [34], IdentiFlight [35], BirdVision and Airelectronics [36] have already implemented and validated their solutions, see Table 1.

Most of the available solutions on the market are based on the monoscopic approach installed on the wind turbine. Only [35] applies stereo-vision installed on a separate tower. However, the system’s orientation is unidirectional. Depending on the sensor used, the detection ranges of the solutions presented vary between 300 m and 1500 m. Most of them use sounds and turbine stopping for collision prevention. It is only the Identiflight solution that consists of an embedded a classifier allowing classification of three different species.

### 2.3. Identification Algorithms

The core of a vision-based system is a detection algorithm. With the growing capability of computers, the AI-based detection algorithm are becoming more efficient [26]. Since 2012, when Krizhevskys’ Neural Network (NN) won the ImageNet competition [37], AI-based solutions have become common for image identification tasks [38]. Furthermore, Machine Learning (ML) and Deep Learning (DL) techniques are being applied for object detection [39], image classification [40] and sound recognition [41].

Regardless of the image sensor used, it is challenging to distinguish birds from other flying objects such as insects, drones, or airplanes. Therefore, the Deep Learning approach is deemed as a suitable tool for bird identification [4,28,42,43,44,45,46]. The comparative analysis of AI-based methods used for bird identification is presented in Table 2.

There are many types of Convolutional Neural Networks (CNN) used for bird identification [50,51,52]. In general, except the number of layers, the important parameters of CNN architecture are: the pooling method, the activation function and the optimization method. It has been found that the pooling with a max 2 × 2 feature window is commonly used. Among the activation functions the ReLU and softmax functions are the most popular. In the training process of the CNN, the Stochastic Gradient Descent algorithm is used as an error minimization method. Other parameters such as input image size, number of epochs, and size of the training dataset, are very individual and are selected for each task respectively.

There have also been attempts to apply identification methods other than by Neural Network, such as Haar Feature Based Cascade Classifier [48], which could give a better individual detection performance in comparison to CNN, but does not perform as well when tasks are conducted on many features [42]. Another method is the Long Short-Term Memory (LSTM) [45], which gives a better performance in bird identification near the moving blades of wind turbines. Nevertheless, identification of small birds with CNN-LSTM still requires improvement. Dense CNN [48] reported good identification performance with additional skip connections [46,53], which improve feature extraction. After 100 epochs, CNN with skip connections reach near 99% identification accuracy, when the same CNN architecture without skip connections reaches 89%.

## 3. Problem Statement, Objectives and Main Contributions

As the survey of related works shows, there are several solutions for bird protection at wind farms. Most of them are based on a single camera, however, with monoscopic vision, it is neither possible to accurately estimate a bird’s size nor its distance from a turbine. Those features are crucial for a reliable and efficient bird collision avoidance system, where the reduction of unnecessary turbine stopping is desired. To stop the wind turbine safely, a bird needs to be detected from a distance up to 200 m–400 m, depending on the species and their flying characteristics. The detection range can depend also on a wind farm’s surroundings and on local environmental authority requirements regarding safety. Most of the protected birds are of medium and large sizes, with a wingspan of more than 1.2 m. Reliable detection of avifauna and its classification is a challenge especially for relatively long distances and varying weather conditions.

The main objective of the paper is to find a structure for a vision-based bird collision avoidance system. The solution should detect and identify a bird from a range of at least 300 m and then classify it into one of three bird categories: small, medium and large. The system should work in real time to ensure that it is possible for the turbine to stop in enough time to avoid collision. The mechanical structure of the system must facilitate its installation on a wind turbine. The system needs to be customizable to adjust its functionalities with respect to requirements of both the local environmental authorities and the wind farm developers. Moreover, the system should assure a high reliability of detection, identification and classification without compromising the needs of low purchase cost along with installation and maintenance costs. To assure a real-time operation mode, the proposed solution applies distributed computing into the IoT paradigm [54]. With a stereoscopic vision acquisition system, AI-based identification and size classification algorithms. The modular structure of the system is proposed to monitor and track bird presence all around the wind turbine. Furthermore, based on processed information about bird category and its distance to the turbine, the system provides a suitable reaction to avoid its collision with the turbine. The proposed decision-making system combines information from each detection module, estimates the bird’s position, classifies it to one of three categories and takes suitable deterrent measures such as stroboscopic lights and/or pulsing sound or stopping the turbine. To design a system, which would meet the requirements specified by the local environmental authorities, wind farm developers and turbine manufacturers, a User-Driven Designed (UDD) methodology [55] is used. The proposed system was implemented, and its prototypes’ performance was experimentally validated and then verified by ornithologists in a real environment on the wind turbine.

## 4. Design

The needs definition and system design phases of the Bird Protection System (BPS) was based on User-Driven Design methodology [55]. In each step of the systematized design process, the following stakeholders are involved: wind farm owners and environmental authorities, future users, ornithologists or wind farm employees preparing reports about bird activities, and finally designers and manufacturers. Such an approach allows for minimization of the risk of not meeting expected needs and allows for a market tailored design solution.

The design of a bird protection system is complex due to the possible counteractive requirements of the stakeholders and environmental authorities. On the one hand, the environmental authorities require high reliability of collision prevention and thus wind turbine stopping on each rare or big bird occurrence. On the other hand, wind farm developers and operators need to prevent unnecessary breaks in power production and wish to minimize turbine-off time.

In Table 3, the functionalities expected of a system and the constraints, which limit possible solutions are shown. For the environmental authorities, wind farm developers and designers along with manufacturers, the overriding goal is to protect birds. Environmental authorities especially, expect high protection of rare birds and additional protection for other birds. Wind farm owners aim to meet the requirements of the authorities while maintaining the highest possible production with the least possible stoppage of turbines. The designers and manufacturers need to create systems that meet requirements of both contributors.

For detection and protection of the birds, the environmental authorities require that a monitoring system will work effectively during the daylight, because most birds do not fly at night [56]. For reliable collision avoidance in the form of turbine stopping is demanded for all the rare species and most big birds of wing spawn larger than 1.5 m. For medium and small birds, deterrent methods such as sound and light signals are allowed from long distance.

Recorded photos and video of each event need to provide data validation and be used as an assessment tool for the prevalence of individual bird species. Moreover, the resolution of the photos and videos should allow the identification of the birds on the captured frames.

The wind farm owners expect reliable collision avoidance systems with minimal impact on the turbines and power production. This could be ensured with reliable classification of bird sizes. With precise information about the detected bird’s size and, knowing its distance, it is possible to minimize turbine stopping only for rare bird species at close distances. Moreover, in some cases stakeholders require additional deterrent methods launched in advance to force the bird to change the flight path before reaching the turbine stopping zone.

As a nonfunctional requirement stakeholders require non-invasive installations, which will not affect the turbine lifetime and its warranty. Easy access to the data gathered by the system e.g., through web and/or mobile applications is crucial for all the stakeholders. Moreover, all gathered data are expected to be retained for at least two years raising the possibility of their presentation and aggregation, which will be useful in annual reports on bird activity.

It is important to develop easy to install and quick to run systems, which in the case of malfunction will be easy to replace. The expected lifetime of the system is to exceed 20 years. It is also crucial to offer a solution that is compatible with existing systems in the turbine, especially in the case of turbine stopping routines. In the case of system installations on different farms, in different countries on different continents it is also necessary to have a remote connection with the system for maintenance purposes and daily status checks.

To achieve a good reception of the system, it should be highly reliable in bird detection with a small number of false positive detections caused by non-bird objects such as airplanes, clouds or insects.

**Figure 1:**
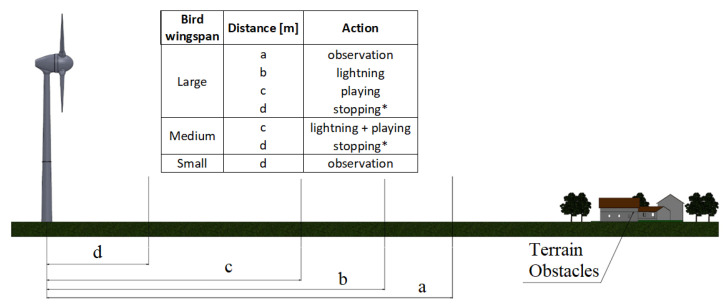
Configuration and variables’ definitions of bird protection system, where (*) means an optional prevention method.

The functionalities and constrains shown were assessed as technically feasible. However, the most important among them is the possibility of system customization and configuration. Different wind farms, even within one country, could require different triggers for activating the turbine stopping routine and/or the deterrent signals. The triggers could be defined with the bird size and distance. Therefore, before designing the system, its basic configuration should be established, according to Figure 1. Following environmental authority guidance, some wind farms need to stop the turbine only for birds considered to be *big* with a wingspan of more than 1.5 m. In other cases it is forbidden to use acoustic signals due to the proximity to buildings. In some cases the operators prefer only to use deterrent signals, without stopping the turbine.

## 5. Modelling

**Figure 2:**
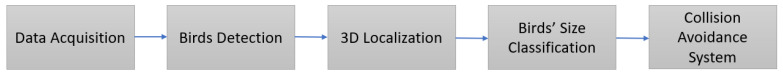
The general system configuration and data processing scheme.

The general system configuration chosen is presented in Figure 2. The system is composed of five separate segments: Data Acquisition, Bird Detection, 3D Localization, Bird Size Classification, and Collision Avoidance System. The Data Acquisition block represents the system hardware and its functionalities, which ensure the reproduction of the bird image onto an image plane. The Bird Detection algorithms allow real-time detection resulting from the object contour. The 3D Localization algorithm is used for estimation of the detected object’s distance and height from the turbine. The object’s contour and its 3D localization from the turbine are used for Bird Size Classification. In the final stage, the Collision Avoidance decision and method is undertaken.

**Figure 3:**
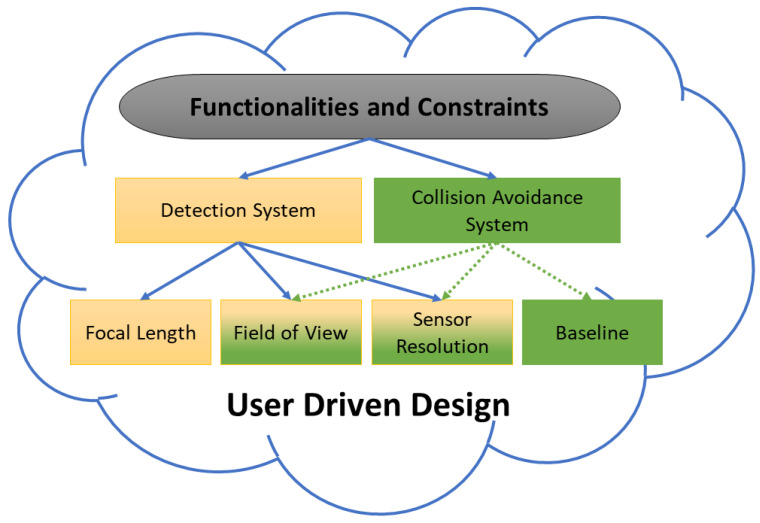
Functionalities and constrains and their impact on hardware components.

To achieve project objectives, the interrelated parameters of the vision system, such as Vision Sensor Size, VSS, Field of View, FoV, and Image Resolution, IR, need to be selected according to system constraints that include cost-efficiency. The inter-dependent parameters of the hardware configuration of the bird protection system are presented in the Figure 3.

To optimize the solution we use the systematic approach presented in this section. In Section 5.1 the parameters of the hardware system components are selected in such a way that focal length, *f*, FoV and VSS are optimal. Then in Section 5.2, the stereo-vision baseline, which ensures bird localization in the range of 300 m is selected. In Section 5.3 the system processing architecture is presented.

### 5.1. Acquisition and Detection System

**Figure 4:**
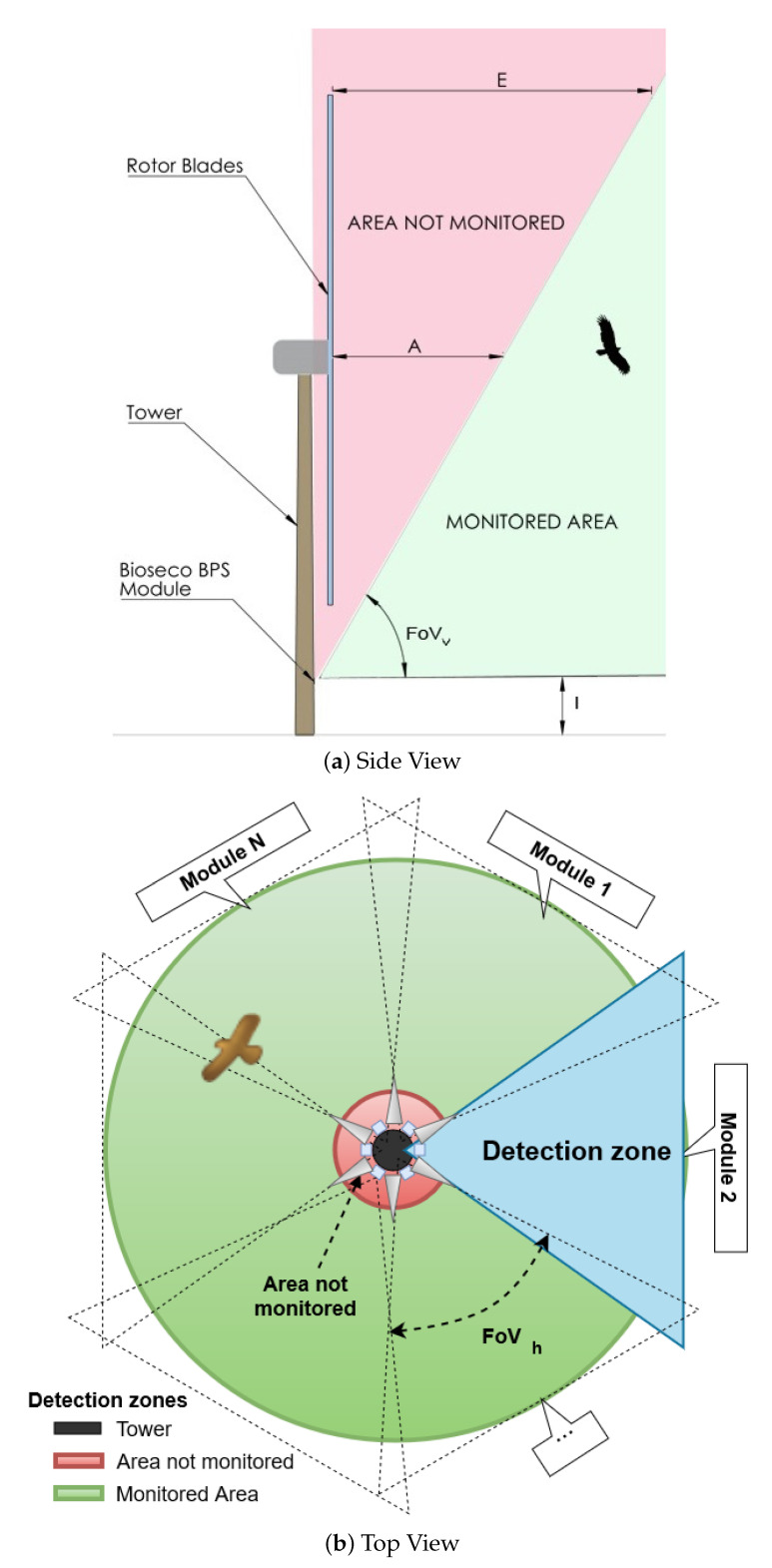
Monitoring area of the system.

As the system needs to monitor space around the turbine, a 360${}^{\circ }$ horizontal field of view ($FoV_{h}$) is required. This can be met by multiplication of the single detection module, as presented on the Figure 4. Furthermore, to prevent a bird’s collision with the rotor blades, the detection system needs to monitor and detect objects in the space in front of the blades at a distance, which allows time for suitable avoidance action.

Overall, the shape of the monitored space depends on the height of system installation *l*, camera vertical $FoV_{v}$ and number of modules used *N*, see Figure 4. To maximize cost-efficiency, the number of modules needs to be limited to $N_{max}=10$, which defines the camera minimal $FoV_{h}$ as:

(1) $$FoV_{h}\geq \frac{360^{\circ }}{N_{max}}\Rightarrow FoV_{h}\geq 36^{\circ }$$

From the *Side View* shown in Figure 4a, the dead zone of the system is defined by the two variables: the dead zone maximal distance *E*, and the blade rotation diameter $R_{B}$. Knowing that the detection range needs to be not less than 40 m to allow time for a successful repelling action, and assuming the maximal height of wind turbines to be around 100 m in height with blade rotation diameter of around 80 m, then the minimal required $FoV_{v}$ can be calculated using formula:

(2) $$FoV_{v}\geq 90^{\circ }-\arctan\left(\frac{E}{R_{B}}\right)\Rightarrow FoV_{v}\geq 63^{\circ }$$

A vision system is defined by the focal length, *f* [m], Vision Sensor Size, $VSS_{h/v}$ [m], Vision Sensor Resolution, $VSR_{h/v}$ [px], and Pixel Size, $p_{W/H}$ [m], which can be different in horizontal (h) and vertical (v) axis. The relationship between *FoV*, VSS and *f* can be shown as:

(3) $$FoV_{h/v}=2\times \arctan\left(\frac{VSS_{h/v}}{2f}\right)$$

Knowing the distance $D_{b}$ [m] of the object to the vision system, the object’s size in width $Size_{W}$ [m] and in height $Size_{H}$ [m] can be estimated using formula:

(4) $$Size_{W/H}=D_{b}\times \frac{p_{W/H}}{VSR_{h/v}}\times \frac{VSS_{h/v}}{f}$$

where $p_{W}$ [px] and $p_{H}$ [px] are the object’s width and height on the image in pixels, respectively. During the simulations we assume that the bird is localized perpendicular to the camera as with during e.g., gliding flight. Therefore, the $p_{W}$ represents the size of a wingspan and $p_{H}$ represents bird length.

From the thumb rule, to detect the bird from the background, its size needs to have at least $p_{W_{min}}=12$ px and $p_{H_{min}}=2$ px. This requirement and constraints of the monitored space (1) and (2) lead to the following sets of equations:

(5) $$\left\{\begin{array}{c} \frac{Size_{W}}{D}\times \frac{VSR_{h}}{VSS_{h}}\times f\geq 12\;px\\ 2\times \arctan\left(\frac{VSS_{h_{max}}}{2f}\right)\geq 36^{\circ } \end{array}\right.$$

(6) $$\left\{\begin{array}{c} \frac{Size_{H}}{D}\times \frac{VSR_{v}}{VSS_{v}}\times f\geq 2\;px\\ 2\times \arctan\left(\frac{VSS_{v}}{2f}\right)\geq 63^{\circ } \end{array}\right.$$

Four common vision sensors and their optical parameters are listed in Table 4.

The case study of system performance is carried out for the medium-sized protected bird Red Kite (Latin: Milvus Milvus) (its length is between 0.61 m and 0.72 m and wingspan between 1.40 m and 1.65 m). Using data from Table 4, image width $p_{W}$ and height $p_{H}$ of the bird at the distance of 300 m as well as horizontal ($FoV_{h}$) and vertical $FoV_{v}$ fields of view were calculated for six different lenses, see Table 5.

In Table 5, the cases when all $FoV_{v/h}$ and $p_{W/H}$ fulfill requirements (5) and (6) are in bold. From the four cases in bold only C1 assures the best performance in terms of computational complexity for the cheapest lens of f=3 mm.

**Figure 5:**
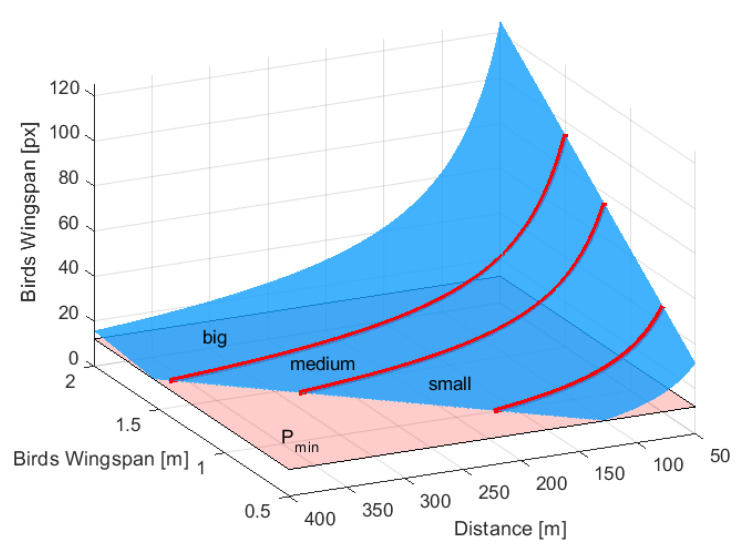
Projection of the bird on an image [px] as a function of bird wingspan [m] and its distance from the baseline, for C1 camera and lens of *f* = 3 mm.

To understand the system performance for the selected C1 camera and lens of f=3 mm, Figure 5 shows how bird projection on the image plane depends on the bird size and its distance from the system. The red lines are demarcations between the three main classes of bird sizes: *big* with a wingspan of more than 1.5 m, *medium* with a wingspan between 1.2 m and 1.5 m and *small* birds between 0.75 m and 1.2 m. The classification capabilities of the system design are represented by the gradient of the blue plane. It can be observed that for a target system range of 300 m, the system is capable of distinguishing between *medium* and *large* birds. However, the *small* bird detection range is below 180 m, which however meets the general system requirements.

### 5.2. Collision Avoidance System

The *Collision Avoidance System* is based on a decision-making algorithm, which processes information about detected object distance and its size. To estimate the object distance from the tower a stereo-vision is used.

**Figure 6:**
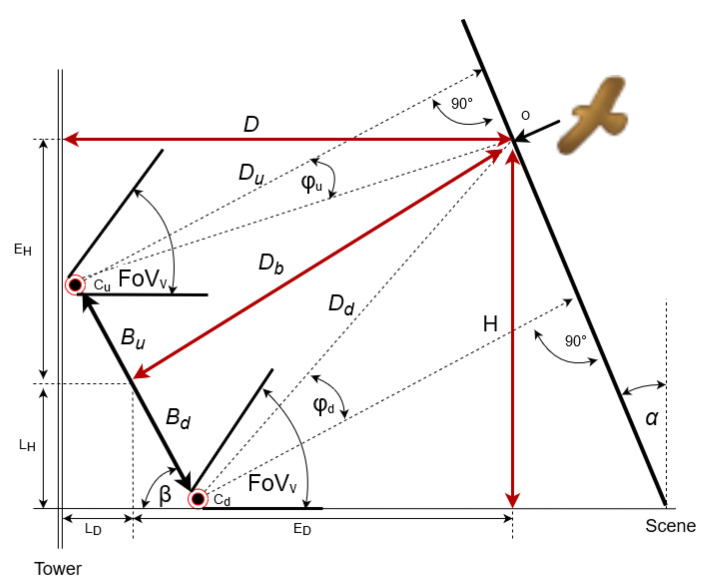
Mapping of stereoscopic camera scenes, defining basic system parameters.

Due to the convenience of installation and maintenance, the vision system is mounted at the lower part of the tower of the wind turbine. Therefore, to cover the required observation area at the front of the blades, the baseline of the stereo cameras is in the vertical position and rotated by the $\alpha =FoV_{v}/2$, as it is shown in Figure 6. By analogy, the stereoscopic scene is also rotated by α. The distance $D_{b}$ between the baseline and the object can be calculated from stereoscopic imaging using the formula [57]:

(7) $$D_{b}=\frac{B\times VSR_{h}}{2\times \left(y_{U}-y_{D}\right)\times \tan\left(\frac{FoV_{v}}{2}\right)}$$

where $\left(y_{u}-y_{d}\right)$ is the difference in pixels between the projections of the object on the *upper* $y_{u}$ and *lower* $y_{d}$ camera matrix respectively, and the baseline $B=B_{u}+B_{d}$ where [58]:

(8) $$B_{u}=D_{b}\times \tan\varphi_{1}$$

(9) $$B_{d}=D_{b}\times \tan\varphi_{2}$$

The distance *D* between the object and the tower, and the height *H* of the object with respect to the lower camera could be calculated from:

(10) $$D=L_{D}+E_{D}$$

(11) $$H=L_{H}+E_{H}$$

The components of (10) and (11) can be found from:

(12) $$\left\{\begin{array}{c} E_{D}=D_{b}\cos\alpha \\ L_{D}=B_{u}\sin\alpha =D_{b}\tan\varphi_{u}\sin\alpha \end{array}\right.$$

(13) $$\left\{\begin{array}{c} E_{H}=D_{b}\sin\alpha \\ L_{H}=B_{d}\cos\alpha =D_{b}\tan\varphi_{d}\cos\alpha \end{array}\right.$$

where $\varphi_{u}$ and $\varphi_{d}$ are angles between the optical axis of the camera and the line section connecting centers of the camera with the detected bird for upper and lower camera, respectively. They could be calculated as:

(14) $$\tan\left(\varphi_{u}\right)=\left(\frac{2y_{u}}{VSR_{h}}-1\right)\tan\left(\frac{FoV_{v}}{2}\right)$$

(15) $$\tan\left(\varphi_{d}\right)=\left(\frac{2y_{d}}{VSR_{h}}-1\right)\tan\left(\frac{FoV_{v}}{2}\right)$$

Using (10)–(15), the distance *D* and height *H* can be calculated from:

(16) $$\begin{array}{cc} D & =D_{b}\tan\varphi_{u}\sin\alpha +D_{b}\cos\alpha =\\ & =\frac{B\times VSR_{h}}{2\times \left(y_{u}-y_{d}\right)\times \tan\left(\frac{FoV_{v}}{2}\right)}\times \left[\left(\frac{2y_{u}}{VSR_{h}}-1\right)\tan\left(\frac{FoV_{v}}{2}\right)\sin\alpha +\cos\alpha \right] \end{array}$$

(17) $$\begin{array}{cc} H & =D_{b}\tan\varphi_{d}\cos\alpha +D_{b}\sin\alpha =\\ & =\frac{B\times VSR_{h}}{2\times \left(y_{u}-y_{d}\right)\times \tan\left(\frac{FoV_{v}}{2}\right)}\times \left[\left(\frac{2y_{d}}{VSR_{h}}-1\right)\tan\left(\frac{FoV_{v}}{2}\right)\cos\alpha +\sin\alpha \right] \end{array}$$

The distance *D* is a non-linear function of VSR, *B*, and FoV. For the chosen parameters of $VSR_{h}$ and $FoV_{v}$, the baseline length, *B* determines uncertainty of distance measurement, which could be estimated using the exact differential method expressed by the formula [59]:

(18) $$\begin{array}{cc} \Delta D_{b} & =\pm \frac{D_{b}}{2\times (y_{u}-y_{d})} \end{array}$$

(19) $$\begin{array}{cc} \Delta D & =\pm \frac{1}{2\times (y_{u}-y_{d})}\times \left[D+B*\sin\left(\alpha \right)\right] \end{array}$$

(20) $$\begin{array}{cc} \Delta H & =\pm \frac{1}{2\times (y_{u}-y_{d})}\times \left[H+B*\cos\left(\alpha \right)\right] \end{array}$$

**Figure 7:**
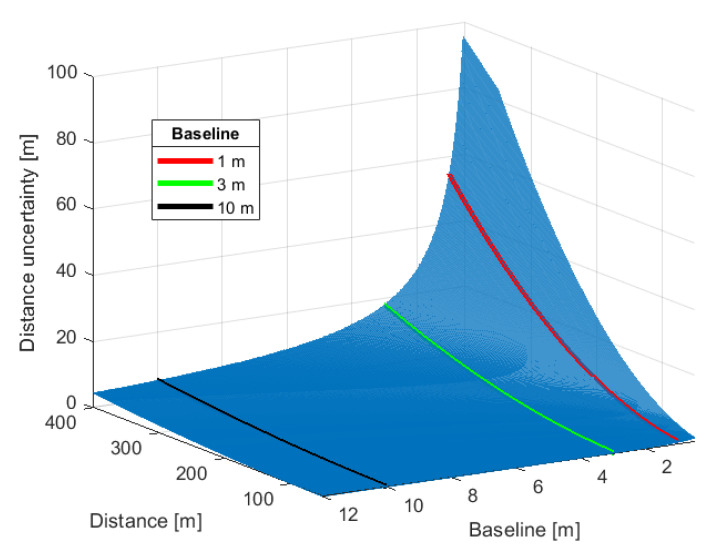
Baseline [m] and distance [m] impact of uncertainty of distance measurement [m]. Black and green lines denote recommended sizes of Baseline of 10 m and 3 m respectively, red line is selected trade-off of 1 m.

**Figure 8:**
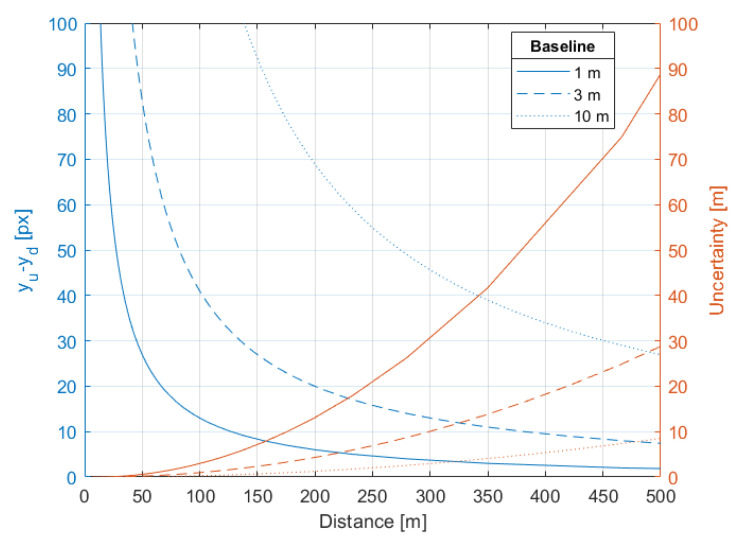
Relationships between object Distance [m] and the difference in pixels on an image [px] (blue color) and a resolution of distance measurement [m] (brown color) for baseline of 1 m, 3 m and 10 m.

Since the desired object detection range is up to 300 m then the recommended baseline should be between 3 m and 10 m [57]. However due to other reasons, such a large baseline is not technically convenient, therefore 1 m baseline is applied. The impact of the baseline on the distance measurement uncertainty is presented on Figure 7 and Figure 8. The figures show how the measurement uncertainty and $y_{diff}$ vary in respect to distance for three values of baseline and for a given worst case of $\phi_{u}$ i.e., $y_{u}=VSR_{v}$, for camera c1 with 3 mm lens. The quantization error for *B* = 1 m is up to three times greater than for *B* = 3 m, but it is still acceptable since at the 300 m distance, the measurement uncertainty is around 30.7 m, which is less than desired 90% accuracy.

**Figure 9:**
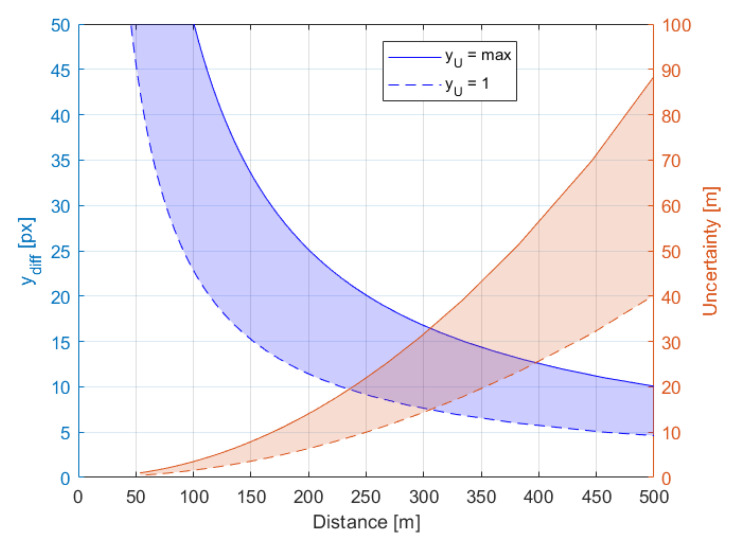
The measurement resolution uncertainty [m] and pixel difference value $y_{diff}$ [px] with respect to distance, for boundary values of the row number of the object projection on the image plane.

**Figure 10:**
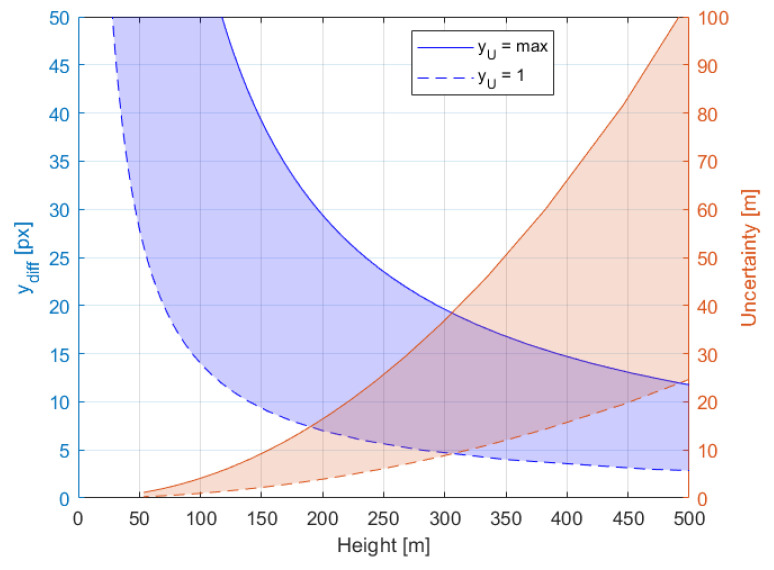
The measurement resolution uncertainty [m] and pixel difference value $y_{diff}$ [px] with respect to height for boundary values of the row number of object projection on the image plane.

The measurement resolution uncertainty and pixel difference value $y_{diff}$ with respect to distance and height for the applied 1 m baseline is shown in Figure 9 and Figure 10, respectively. To see how the pixel difference value $y_{diff}$ and uncertainty depends on angles $\varphi_{u}$ and $\varphi_{d}$, the values of the $y_{u}$ and respective $y_{d}$ are set to minimum and maximum i.e., 1 px and $VSR_{v}$, respectively. The distance estimation uncertainty of the desired detection range of 300 m varies from 15 m to 33 m, for $y_{diff}$ equals 7 px and 17 px, respectively. The uncertainty of object height measurement is greater than for distance measurement and at the desired detection range of 300 m varies from 9 m to 36 m, for $y_{diff}$ equals 5 px and 19 px, respectively.

### 5.3. Processing System Architecture

**Figure 11:**
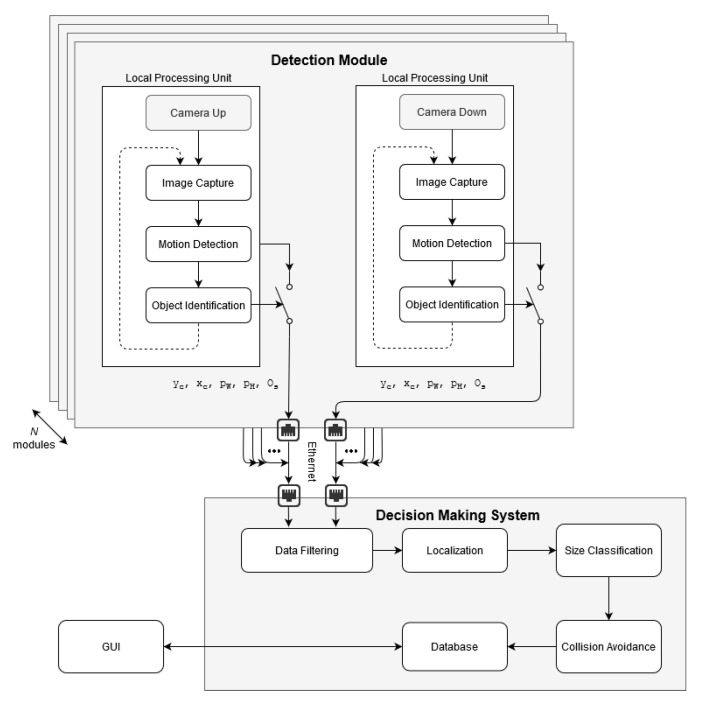
Illustration of system general processing architecture.

The system processing architecture is shown in Figure 11. To ensure real-time performance, the system is based on the distributed computing concept. It consists of two main subsystems: Detection Module and Decision-Making System. The first one uses embedded CPU and GPU architecture of the Local Processing Unit. However, data processing at the second subsystem is performed at the database server. The input data of the Detection Module are provided from the stereo-vision system, consisting of two integrated cameras. Data from each camera is used for independent Motion Detection and Object Identification.

When a moving object is identified as a bird, a trigger is activated and the information determined by the Motion Detection algorithm about objects size $o_{s}$ [px], its width $p_{W}$ [px] and height $p_{H}$ [px], and the geometric center coordinates $x_{c}$ and $y_{c}$ are sent to the Decision-Making System. Additionally, the frame with the identified bird is received by the Decision-Making System for data handling. The Local Processing Unit is designed applying the Internet of Things (*IoT*) concept with IP addressing, so the Decision-Making System could easily identify the source of the data stream. Information from the Local Processing Units combines estimation of the object’s distance with classification of the bird size. Based on the classification a decision about the action to be performed by Collision Avoidance is taken. All data are stored on the Database and available on the website via GUI.

### 5.4. Detection Module Processing

**Figure 12:**
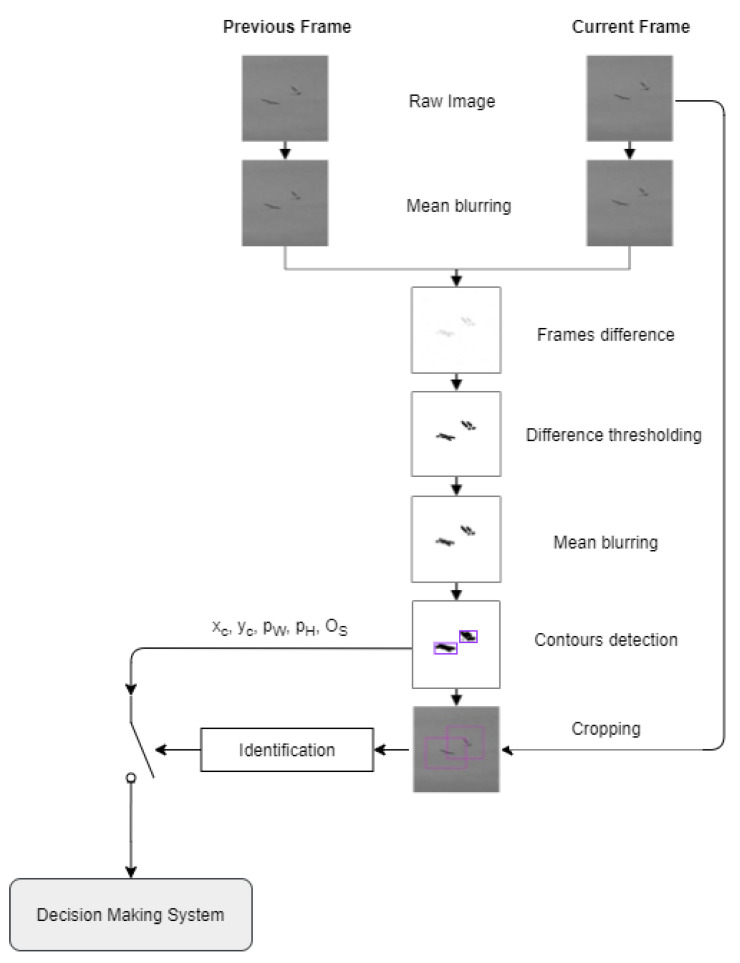
Bird detection algorithm flowchart illustrated by original images from the system.

The bird detection algorithm presented in Figure 12 is based on motion detection, which guarantees low computational complexity needed for real-time processing. First, two image frames, current and previous, are subjected to Mean blurring using the Gaussian blur method, also known as Gaussian smoothing. This step aims to minimize the impact of small lighting changes. Then, the frames are subtracted from each other to determine differences between the images, caused by an object’s movement. Frames difference generates the gradient matrix containing the value of the difference in each pixel. To filter out negligible differences, the image is subjected to Difference thresholding At the resulting image, the moving object could appear as two-fold. To determine the object’s singular envelope, the split images need to be merged, which is done by the Mean blurring using Gaussian blur filtering and Binary thresholding Then Contour detection is applied on the resulting binary image to get the envelope of the object. Knowing the object contour, the value of $p_{W}$ and $p_{H}$ can be calculated using the center of mass of the object contour and image moments [60]. If the object is smaller than $p_{W_{min}}=12$ px or $p_{H_{min}}=2$ px, it is neglected as an artefact. Otherwise, objects smaller than 100 px, are cropped using a standard mask of 100×100 px. If the object is greater than 100 px in any $p_{W/H}$, then the cropping mask is resized to a greater value of $p_{W}$ or $p_{H}$. The object size, $o_{s}$, is calculated as the number of pixels over the contour, which is computed using the Green formula [61]. The object size is used in the Decision-Making System for object classification.

In the next step, the cropped image is subjected to the identification process since the Motion Detection algorithm determines all moving objects, not only birds, but also insects, planes, drones or moving clouds. The applied Convolutional Neural Network, CNN, was selected as a standard for object classification.

**Figure 13:**
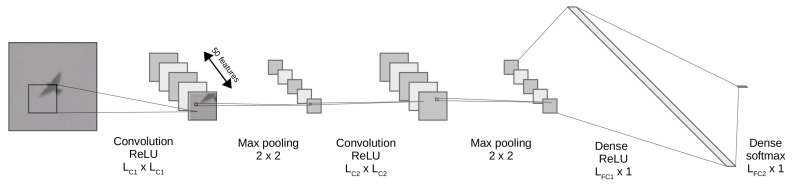
Architecture of Convolutional Neural Network used for bird identification.

The architecture of the proposed CNN is presented in Figure 13. It consists of two convolutional layers with sizes of $L_{C1}$ and $L_{C2}$, which are used for feature extraction. Two additional fully connected layers $L_{FC1}$ and $L_{FC2}$ are responsible for class probability calculation and final object identification. The layer $L_{FC1}$ uses the *Softmax* function to obtain binary information *bird/not bird*. The layers $L_{C1}$, $L_{C2}$ and $L_{FC1}$ are activated by the *Rectified Linear Unit* function. Between layers $L_{C1}$, $L_{C2}$ and $L_{FC1}$, the *Max pooling* with 2×2 pool size is used. Thus, the number of features was set to 50.

At the first stage of CNN design, its model needed to be optimized and trained. The aim of the optimization process is to select a suitable number of neurons in each layer ($L_{C1}\times L_{C2}\times L_{FC1}$) ensuring real-time inference with high reliability. The size of $L_{FC2}$ for the binary decision-making task was á priori chosen for 2 and the *validation split* was set to 10%. Parameter ϵ of ADAM optimizer was set to $10^{-7}$, *learning rate* to $10^{-5}$, *training length* was set to 50 epochs. The 3×3 convolution kernel was used.

**Figure 14:**
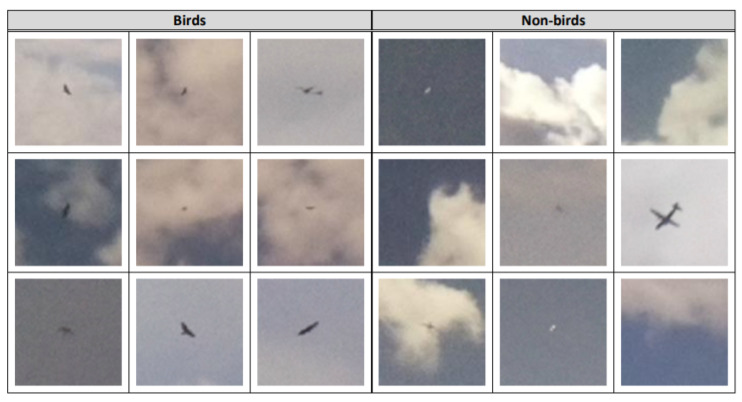
Examples of images used for the training process.

For the CNN training, a database of 45,000 *birds* and 45,000 *non-birds* RGB images previously identified by the Detection algorithm were used. All images were manually double-checked to ensure best quality of the training process. The *birds* dataset consists of images of different species of *small*, *medium* and *large* birds taken at a distance of 40 m to 500 m from the wind turbine. However, the *non-birds* dataset consists of any other objects identified by the *Detection algorithm*. Objects bigger than 100 px × 100 px were re-scaled to Region of Interest (ROI). The examples of images used in CNN training are shown in Figure 14. The CNN was trained using 2 × NVIDIA Quadro RTX6000+ NVLink and Intel Xeon W-2223 3.6/3.9 GHz with 128 GB DDR4 ECC.

To optimize the CNN, it needs to be quantitatively evaluated in respect to the quality of the identification process. The following parameters were selected: *Precision*, *Recall*, *F*1, *Specificity* and *Identification accuracy*, as the most commonly used [62].

*Precision*, also called Positive Predictive Value is the ratio of correctly identified birds to all objects identified as birds. It is a measure of confidence that an identified object is truly a bird:

(21) $$Precision=\frac{TP}{TP+FP}\;,$$

where *TP* stands for the number of *True Positive* detections, and *FP* means the number of *False Positive* detections.

*Recall*, also known as *Detection Sensitivity* is the ratio of correctly identified birds to all birds included in the test set. This parameter is a crucial measure of birds missed by the system:

(22) $$Recall=\frac{TP}{TP+FN}\;,$$

where *FN* is the number of *False Negative* detections.

The *Harmonic Mean*, *F*1, combines *Precision* and *Recall* into one coefficient, which is a measure of correctly identified birds to all false detections, both positive and negative:

(23) $$F1=\frac{2\times Precision\times Recall}{Precision+Recall}=\frac{2TP}{2TP+FN+FP}\;.$$

*Specificity* is the ratio of correctly identified non-birds to all non-birds included in the test set. The parameter is also a measure how many non-birds were miss-identified by the system as birds:

(24) $$Specificity=\frac{TN}{TN+FP},$$

where *TN* is number of *True Negative* detections

*Identification Accuracy* is the ratio of correctly identified birds and non-birds to all objects in the test set. This is also a measure of system reliability to distinguish between birds and non-birds:

(25) $$Accuracy=\frac{TP+TN}{\left(TP+TN)+(FN+FP\right)}$$

To optimize the CNN, performance evaluation was carried out using a test dataset of 40,000 *birds* and 40,000 *non-birds* images. The results are presented in Table 6. For each simulation, the Feed-Forward, *FF*, time was estimated on the NVIDIA Quad-core ARM Cortex-A57 MPCore processor. This parameter was calculated as a mean time of FF process over each of 80,000 testing images.

The fastest solution, with the *FF* time bellow 1 ms, is when CNN contains $L_{C1}$ = 32, $L_{C2}$ = 32 and $L_{FC1}$ = 32 neurons. However, the obtained *Precision* and *Specificity* were the lowest of the tested parameters. The greatest *Precision*, *F*1 and *Accuracy* are for CNN of $L_{C1}$ = 32, $L_{C2}$ = 64 and $L_{FC1}$ = 256 neurons. However, for this case, the *FF* time of 2.85 ms could make impossible the real-time performance. Therefore, the CNN consisting of $L_{C1}$ = 32, $L_{C2}$ = 32 and $L_{FC1}$ = 128 neurons with FF = 1.09 ms has been selected as a reasonable trade-off.

The *Recall* values shown in Table 6 are the same for each tested CNN. With the variation of 0.002, this coefficient is statistically insignificant. It could mean that the birds differ strongly from other objects and could be easily distinguished by the tested CNNs. Overall, the system can assure low *FN* detections, which is desirable for safety applications.

### 5.5. Decision-Making Module Software

**Figure 15:**
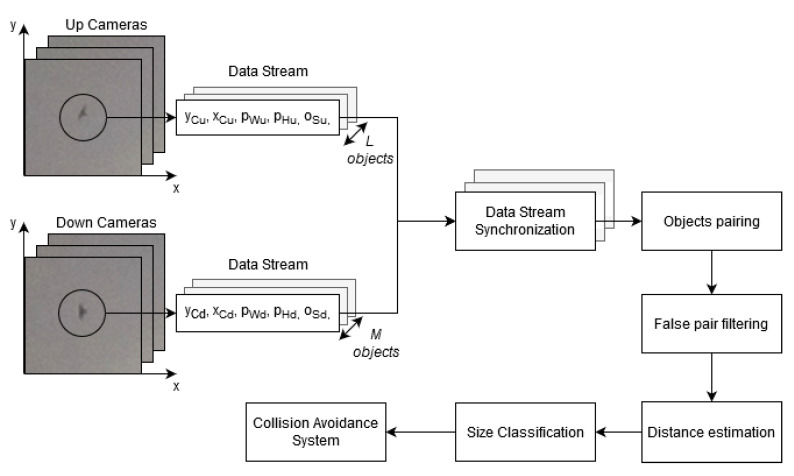
Block diagram of decision-making system.

The block diagram of the Decision-Making System is presented in Figure 15. This system combines information from all Local Processing Units installed over the Wind Turbine. The applied distributed computing configuration of the system along with the IoT technology facilities allow for real-time performance of up to 20 Detection modules.

The Decision-Making System works on data-sets generated by the Local Processing Unit from the *Upper* and *Lower* cameras. The Data Stream containing information about the bird’s size $o_{s}$ [px], its image width $p_{W}$ [px] and height $p_{H}$ [px] along with the image geometric center coordinates $x_{c}$ and $y_{c}$. First, the data is stored and then processed at the Data Stream Synchronization block, responsible for merging two data-streams from each Local Processing Unit. Based on the timestamp, the data from the upper and the lower cameras are fused. The identified objects’ coordinates are paired based on *Geometric distance matching* by minimization of the difference in objects image centers’ coordinates in the *X* and *Y* axis, using the following formula:

(26) $$\underset{\begin{array}{c} {}_{k\leq \min(L,M)}\\ {}_{k\in N_{+}} \end{array}}{⋀}o_{c_{diff}}\left[k\right]=\underset{\begin{array}{c} {}_{i\leq L}\\ {}_{j\leq M}\\ {}_{i,j\in N_{+}} \end{array}}{⋀}\min\left(\sqrt{{\left(x_{c_{u}}\left[i\right]-x_{c_{d}}\left[j\right]\right)}^{2}+{\left(y_{c_{u}}\left[i\right]-y_{c_{d}}\left[j\right]\right)}^{2}}\right)$$

where *i* and *j* are the indexes of *L* objects identified at the upper and *M* objects identified at the lower camera, respectively. For each object at the upper camera, the geometric distance to each object at the lower camera is calculated, and then a set of *K* = min*(L,M)* pairs with minimum distances is taken into further consideration.

Then, the False pair filtering algorithm, removes pairs of maximum center differences in *x* and *y* coordinates greater than 150 px. Such objects could be insects flying close to the system. Meanwhile the minimum of the $y_{c_{diff}}$ is limited to 1 px, since negative value of the distances on such directed stereo-vision cameras is not possible.

When the center points from the 2D image planes of the upper and lower cameras are paired, then in a Distance estimation and classification block, a distance $D_{b_{c}}$ to the object’s center could be calculated using (7) where $y_{c_{u}}$ and $y_{c_{d}}$ are *y* coordinates of the image geometric centers of the upper and lower images, respectively.

Knowing the distance $D_{b_{c}}$ and using information about size of bird images in terms of $p_{W}$ and $p_{H}$, the bird wingspan $P_{W}$ [m] and height $P_{H}$ [m] could be estimated using:

(27) $$\left\{\begin{array}{c} P_{W}=\left(D_{b_{c}}\pm \Delta D_{b_{c}}\right)\times p_{W}\times \frac{VSS_{h}}{f}\times \frac{1}{VSR_{h}}\\ P_{H}=\left(D_{b_{c}}\pm \Delta D_{b_{c}}\right)\times p_{H}\times \frac{VSS_{h}}{f}\times \frac{1}{VSR_{h}} \end{array}\right.$$

From $o_{s}$ [px], which is estimated as the number of pixels over the bird’s contour, the object area $O_{s}$ [m${}^{2}$] could be calculated as:

(28) $$O_{s}=o_{s}\times {\left(\frac{D_{b}}{f}\right)}^{2}\times \left(\frac{VSS_{h}}{VSR_{h}}\right)\times \left(\frac{VSS_{v}}{VSR_{v}}\right)$$

**Figure 16:**
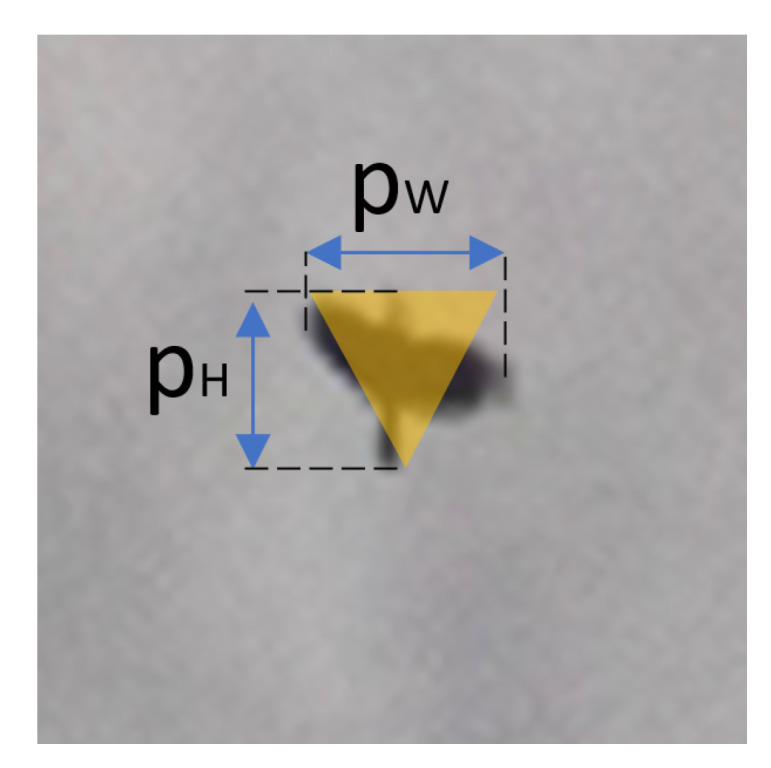
Graphical approximation of the bird’s size calculation.

An isosceles triangle, which is shown in Figure 16 has been used as an approximation method $o_{approx}$ to evaluate system performance. The triangle base corresponds to the bird’s wingspan $P_{W}$ [px]. However, the height of the triangle is equal to the $P_{H}$ [px] and denotes the bird’s height.

(29) $$O_{approx}=\frac{P_{W}\times P_{H}}{2}.$$

Based on the size estimate, the developed classifier distinguishes three bird size classes: *small*, *medium* and *large*. In Table 7, classification boundaries of *small*, *medium* and *large* birds are presented. The *small* birds are these whose wingspan is between 0.65 m and 1.25 m and height is between 0.32 m and 0.39 m. Birds of wingspan between 1.26 m and 1.50 m, and height from 0.40 m to 0.55 m is classified as *medium* birds. The *large* birds have wingspan above 1.50 m and height above 0.55 m.

**Figure 17:**
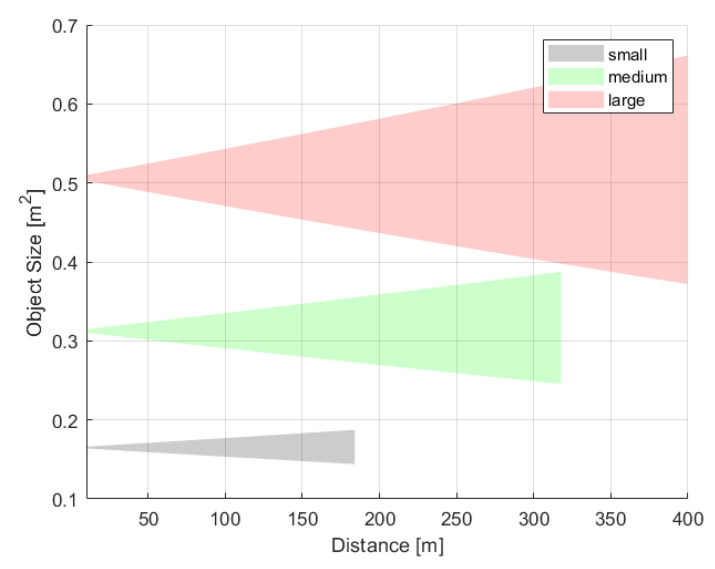
The change of object size $o_{approx}$ with distance caused by the quantization error of distance measurement for average representative of *small*, *medium* and *large* bird.

The representation of the bird on an image plane depends mostly on the object distance from the system. Therefore, the uncertainty of distance measurement impacts mostly on the size classification accuracy. The uncertainty ranges of image sizes for each of the three class average sizes are presented in the Figure 17. Within distance ranges of each class, there are no overlaps for class average sizes. However, the boundaries between classes could be very fuzzy, and therefore the classification could be ambiguous, especially at long distances. To reduce the fuzziness, each object is differentiated based on three parameters, $p_{W}$, $p_{H}$ and $o_{s}$. Due to safety reasons the valid class always selects the largest of the indicated parameters. For instance, if one parameter indicates a *large* bird, then a bird is classified as *large*. Similarly, if even just one parameter indicates a *medium* bird and the two remaining suggest *small* bird, then the bird is classified as a *medium*.

Based on the object’s distance and its size, the Collision Avoidance system activates one of its predefined actions. A user could specify the distance and size category for activation of sound and/or strobe repellents or even turbine stopping. An example of the system setting is presented in Figure 1.

The system includes archiving of undertaken actions, which could be later analyzed by authorities. The archive consists of photos and/or videos. This functionality is required by some stakeholders and users.

## 6. Prototyping and Testing

In this section, the prototype of the system and its installations are described. Furthermore, the system and its implementation have been validated and the test results are shown here.

### 6.1. System Prototype

**Figure 18:**
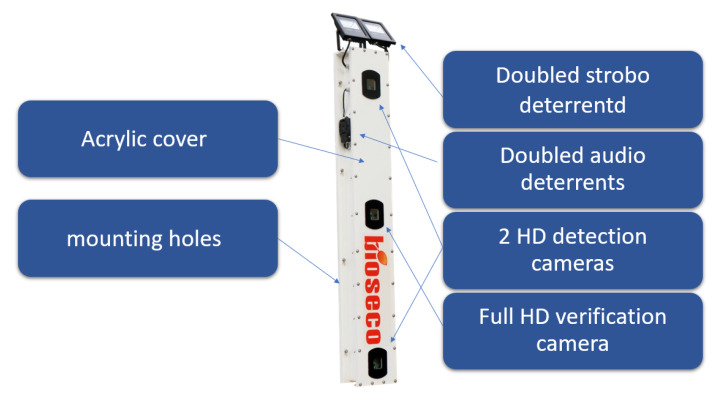
A photo of the detection module of the bird protection system.

The optimized hardware and software have been implemented on suitable platforms to make possible the validation of the system in the field. The prototype of detection modules presented in Figure 18 is composed of two IMX219 cameras with 3 mm lens. An optional full HD camera using an IMX219 sensor is installed for video event verification. As a *Local Processing Unit* a Quad-core ARM Cortex-A57 MPCore processor with 2 GB RAM for *object detection* and 512-core Volta GPU for *object identification* were used. The *Decision-Making System* was implemented on a database Dell server with Xeon X5687 processor of 3.6 GHz and 8 GB of RAM. Two hard drive with 8 TB memory are included for media storage. The connection between the *Decision-Making System* and *Detection Modules* is provided by Ethernet protocol. The *Detection Modules* are powered using Safety Extra-Low Voltage, SELV.

For the *strobe deterrent system*, two 10 W, 800 lm and 4500 K lamps with flash frequency of 5 Hz–7 Hz are applied. As an *audio deterrent*, two 124 dB speakers generating a signal with frequency range of 2.4 kHz–6.5 kHz are used. The sound generated by the audio repellents is randomly selected from the set of sounds prepared by ornithologists. The hardware parameters of the *deterrents system* are selected based on a *survey of related works* and market availability of off-the-shelf products. To ensure low weight, the detection system is made in an acrylic cover.

### 6.2. Implementation and Testing in the Field

**Figure 19:**
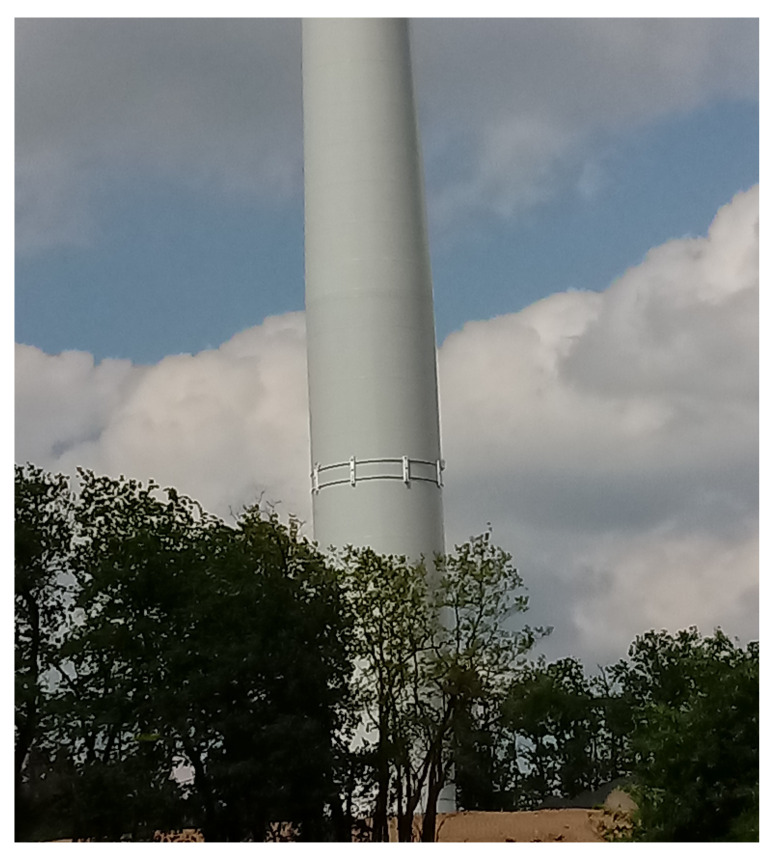
The system prototype mounted on a wind turbine at the test field. The system consists of eight *Detection modules* fixed on the tower wall and the *Decision-making system* placed inside the tower.

The developed system has been installed on the wind turbine at a wind farm in the northern part of Poland as shown in Figure 19. The system is designed to allow a non-invasive installation by using steel clamps fixing the modules to the wind turbine. The detection modules are uniformly distributed around the wind turbine, connected to a server using the IoT concept and powered using low voltage. To allow easy access of the stored data and to monitor the status of the *Detection Modules* in real time, a Long-Term Evolution, LTE, wireless broadband communication is used. The test field was chosen to allow horizontal and vertical coverage of the most crucial area along with good detection range of the system.

**Figure 20:**
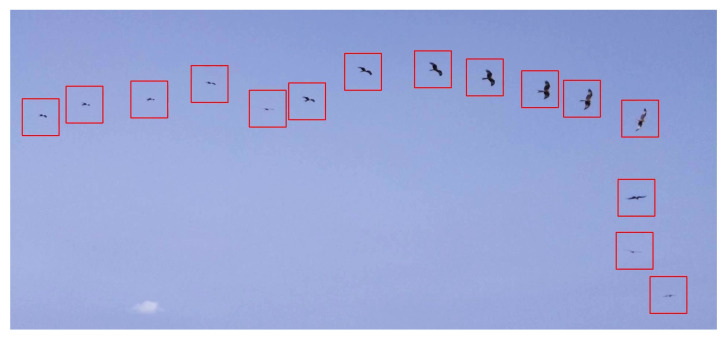
Time-lapse photos of detection samples of Red Kite.

Figure 20 shows samples of time-lapse photos of a Red Kite caught by a detection module. The 2D pictures of the bird vary in size depending on its distance from the system.

### 6.3. Distance Measurement Evaluation

**Figure 21:**
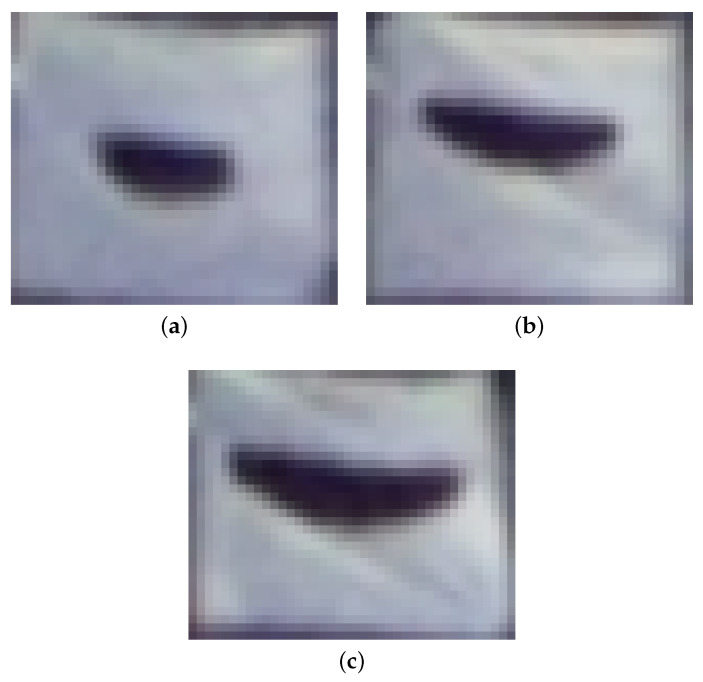
Pictures of three bird silhouettes simulating (**a**) *small* ($P_{W}$ = 0.8 m and $P_{H}$ = 0.3 m), (**b**) *medium* ($P_{W}$ = 1.2 m and $P_{H}$ = 0.4 m) and (**c**) *large* ($P_{W}$ = 1.5 m and $P_{H}$ = 0.5 m) birds at distance of 150 m.

The system’s localization and size classification performance at different distances was validated using birds painted on canvasses. Three bird silhouettes simulating *small*, *medium* and *large* objects are shown in Figure 21. The measurements were taken from 50 m up to 300 m with a step of 50 m. The object’s dimensions of wingspan ($p_{W}$), height ($p_{H}$) and contoured area ($o_{s}$) along with $y_{u}$ and $y_{d}$ were measured to estimate the distances ($D_{b}$) and objects’ *Wingspan* ($P_{W}$), *Height* ($P_{H}$), approximation size $O_{approx}$ and contour size $O_{s}$. The test results are summarized in Table 8. The value of $\Delta D_{b_{ref}}$ is theoretical quantization error for the measured $D_{b}$, while ($D_{b_{ref}}-D_{b}$) is a real measurement uncertainty.

Most importantly, the experimental results show the high measurement repeatability. All tests of *small* size class validated the theoretical model, and measured distances are within quantization range. As expected, an estimation of $P_{W}$ is more accurate then $P_{H}$, since bird’s height is fairly small. An interesting conclusion would be that an approximated size estimation $O_{approx}$ matches real size more precisely than that from the contour. For the *medium* class, experimental results are slightly worse than for the *small* size class, but just for the furthest distance of 250 m, where the accuracy is at a level of ±1 quantum instead of assumed ±1/2 quantum. A similar conclusion at this distance can be drawn for the large size silhouettes. However, for the longest distance of 300 m the measurements are very accurate with an uncertainty of 0.7%. Overall, the distance estimation uncertainty, calculated as $D_{ref}-D$ is less than 5% of the reference distance, which meets user’s expectation of 10% localization accuracy defined in Table 3. The precision of the objects’ *wingspan* and *height* estimation decreases with distance; however, it is still sufficient to distinguish the class of the object. It has been proved that the proposed simplified approximation method of the bird’s size $O_{approx}$ using the isosceles triangle is enough for its estimation.

### 6.4. System Validation

**Figure 22:**
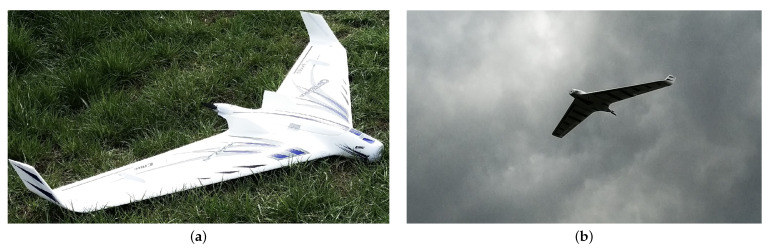
Photos of the fixed-wing drone used for the system validation (**a**) on the ground (**b**) in flight.

**Figure 23:**
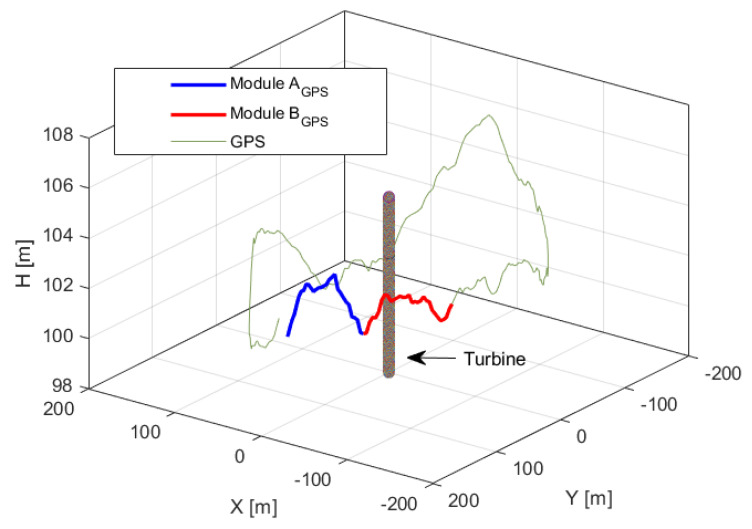
The GPS flight path of the test drone.

For system dynamics validation, a fixed-wing drone was used. The bird-like drone with a wingspan of 1.99 m and height of 1.03 m and weight of 2 kg, see Figure 22, was programmed to fly around the wind turbine at 100 m high and 150 m distance. The drone was equipped with a GPS sensor and autopilot for remote control. The GPS flight path is shown in Figure 23. The GPS measured average flight height and distance from the wind turbine were (102.9 ± 1.5) m (143.3 ± 2.5) m, respectively. Average speed of the drone was 15 m/s. The flight was monitored by the eight modules installed on the wind turbine. In Figure 23, zones of two randomly selected adjacent detection modules are depicted in blue ($ModuleA_{GPS}$) and red ($ModuleB_{GPS}$). The *Module A* detected the drone 42 times, whereas the *Module B* 37 times.

**Figure 24:**
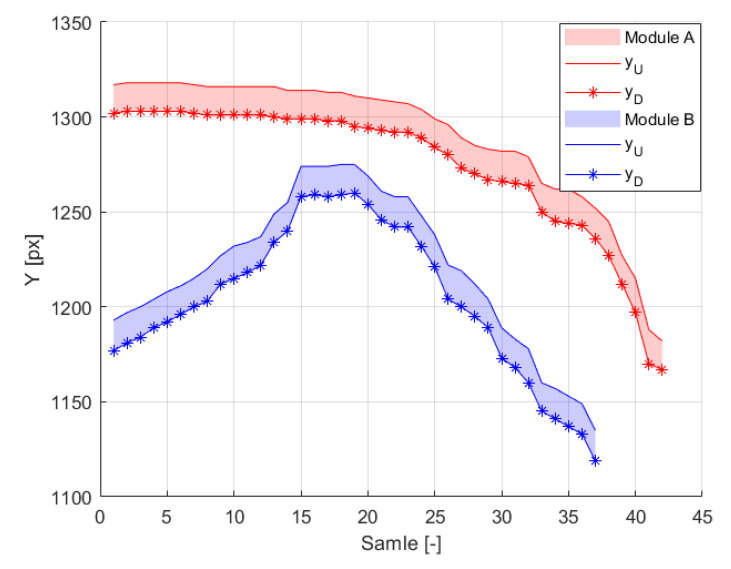
The variation of $y_{u}$ and $y_{d}$ with time (sample) for *Module A* and *Module B* in the drone test.

**Figure 25:**
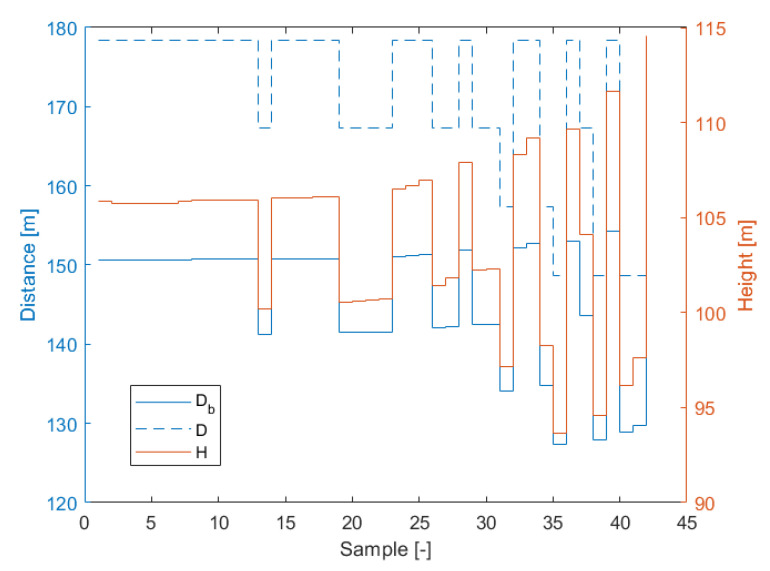
Variation of estimated distances *D*, $D_{b}$ and *H* for *Module A* in the drone test.

**Figure 26:**
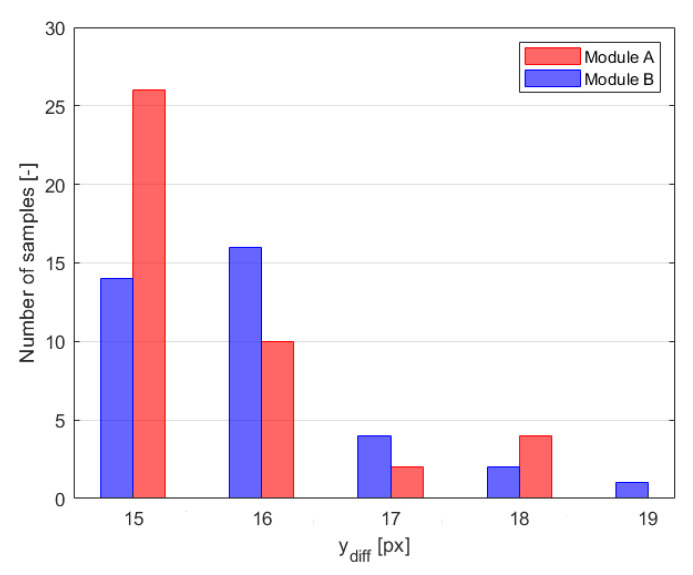
Histogram of variation in pixel difference [px], $y_{diff}$ for module A and B in the drone test.

Figure 24 presents time samples of $y_{u}$ and $y_{d}$, and Figure 25 presents corresponding estimated distances *D*, $D_{b}$ and *H* obtained for *Module A*. The histogram of measured difference in pixels, $y_{diff}$ is presented on the Figure 26. The most frequent measured value of $y_{diff}$ was 15 px and 16 px for *Module A* and *Module B* respectively, which corresponds to $D_{b}$ equal to 178.4 m and 167.2 m, respectively.

**Figure 27:**
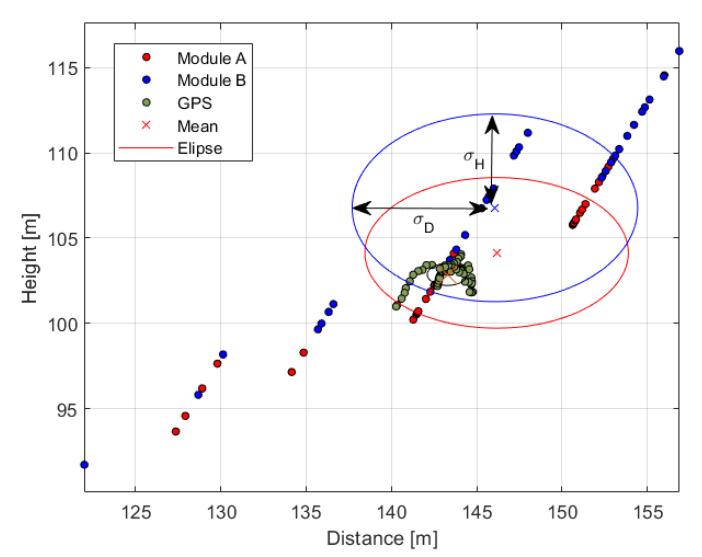
Distance from a wind tower vs height of drone test. Green dots-GPS data. Red and blue dots-data from module A and module B, respectively. Corresponding color ellipses illustrate standard deviations of respective distance and height measurements.

Figure 27 shows samples of estimated distance *D* and height *H* for *Module A*, *Module B* and *GPS* data. On the figure, three ellipses illustrate measurement statistics of each module and GPS. For each ellipse, the center depicts mean value of *D* and *H* while a semi-major axis represents standard deviation $\sigma_{D}$ and a semi-minor axis corresponds to standard deviation $\sigma_{H}$. The maximal error of distance measurement is equal to 12.9 m and 18.2 m for *Module A* and *Module B*, respectively, in distance and 10.4 m and 11.9 m for corresponding height measurement. The test measurement results of heights *H* and distances *D* are summarized in Table 9. The difference in mean values between modules and GPS data is 2.8 m and 2.9 m in distance and 1.2 m and 3.8 m in height for *Module A* and *Module B* respectively. The average values of the measurements are within the range of uncertainties error $\Delta D_{b}$ = 3.85 m.

### 6.5. System Verification by Ornithological Observations

The system verification was carried out by experienced ornithologists at random dates, times and weather conditions. The 67.5 h of observations were conducted for 14 days between May and July 2020. The time, roughly estimated distance and height of birds observed by the ornithologist were recorded. The records were compared with events reported by the system as summarized in Table 10. All Wood Pigeons and Common Buzzards observed by the ornithologist up to 200 m, which is in the range of small bird detection zone were correctly detected by the system. The Wood Pigeons, which belong to the small bird category were correctly classified with a rate of 8/10, while 2/10 were assigned as medium. In case of 10 observed Common Buzzards, whose size lies on the border of small and medium classes, 5 were classified as small and 3 were marked as medium, but 2 were classified as large, while over-sizing is considered to be a less crucial classification mistake.

In the case of medium-sized birds such as Raven and Marsh, the system correctly detected all 26 birds at up to 100 m, and just 1 of 20 was missed in zone of 100 m–200 m. For a distance over 200 m ornithologists observed 29 birds while the system detected 23 of them. As the lowest limit of Raven and Marsh harrier wingspan belongs to the small class, 16 out of 70 birds were classified as small, 23 were considered to be medium and 31 were marked as large. For Herring gull of wingspan covering medium size boundaries, each of the three detected birds was classified to a different class, which can be symptomatic for this class of bird.

Ornithologists observed just six medium/big birds, two Red kites and four Cranes. All of them were detected by the system up to 200 m, but one Crane was missed by the system at a distance of over 200 m. All observed Red Kite were classified as medium, while one of the Cranes was classified as medium and two as large.

**Figure 28:**
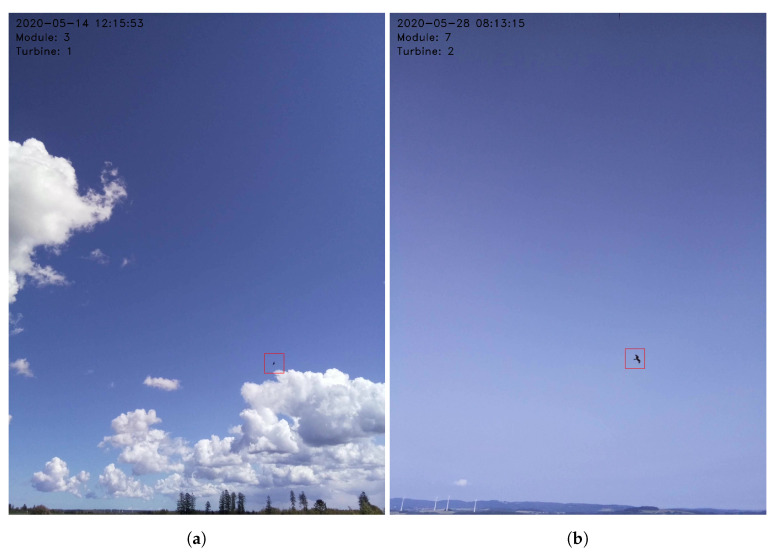
Example images with depicted detected and classified (**a**) Raven, (**b**) Red Kite.

**Figure 29:**
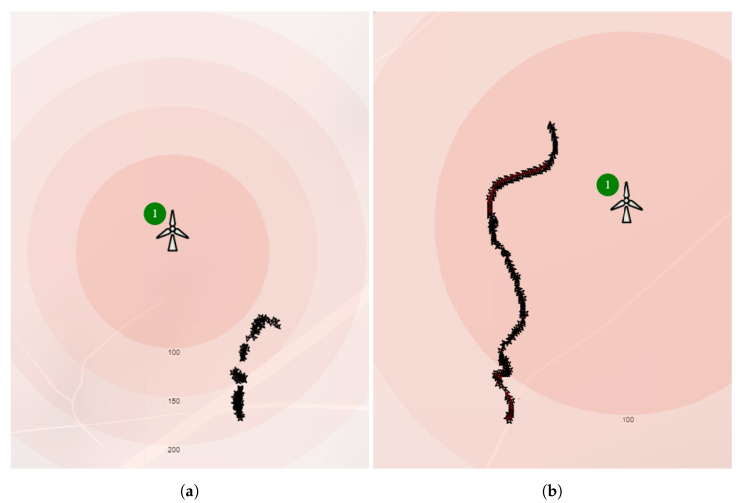
Examples of flight paths of (**a**) Raven, (**b**) Red Kite observed on 21 May 2020 visualized on Google Maps [64].

Overall, the ornithologists identified 105, while the system missed 9 of them, but no bird was missed at a distance below 100 m and one medium size bird was omitted at a distance of between 100 m and 200 m. Most omissions, 7/9 happened for birds observed over 200 m and one of these 7 was a big bird. Furthermore, in one case of 98 bird classifications, the system misclassified a bird into a lower class than expected. The images of detected Raven and the Red Kite are shown in Figure 28a,b, respectively. The 2D flight routes of these birds are shown in Figure 29.

**Figure 30:**
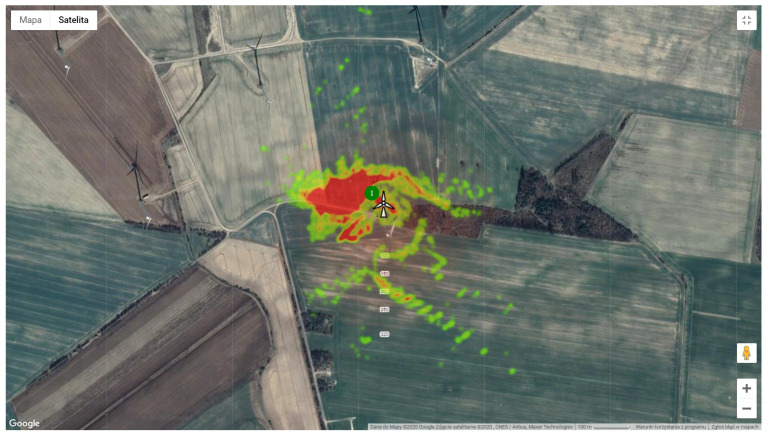
A heat-map of the one-day observation where the red color depicts the highest density of detections.

The Figure 30 shows the top view of bird detection density for one day of ornithologist observations. The map was created using *Google* heat maps layer [64]. The area of higher detection intensity is colored in red, whereas the area of lower intensity appears in green. The majority of detected birds in flight are to the West and North of the Wind turbine. There are also some more distant detections to the East and South.

## 7. Conclusions

This article tackles the problem of avifauna preservation at a wind farm. To reduce bird mortality near the wind turbine, a vision-based collision avoidance system is proposed. To assure real-time operation mode, the proposed solution applies a distributed computing paradigm embedded into IoT methodology. It means that the data processing is split between the *Local Processing Unit* and the *Decision-Making System*. The second one undertakes predefined repelling action based on the prepossessed information of the object position on the images at the top and bottom camera.

The system has been developed using a User-Driven Design (UDD) approach, which assured that the stakeholders, environmental authorities, future users and designers were actively involved in the design process. Such an approach ensured that the designed system was tailored by not only the market, but more importantly by the authorities. Moreover, customization opportunities have also been implemented to increase system adaptability for different installations.

The developed stereoscopic vision acquisition system allows the detection of an object and determines its distance from the turbine and then estimates its size. The designed AI-based identification method and size classification algorithm used for decision-making, reduces false positive detection and limits turbine stopping only for detected rare big birds. The repelling method implemented was designed according to state-of-the-art and has a cascading form composed of light and sound deterrents, which are backed by the most secure collision prevention method: turbine stopping.

The presented stereoscopic vision acquisition system was evaluated by the measurement of bird silhouettes painted on a canvas. The performed tests confirmed the assumed detection, localization and size classification performance quality for *small* birds up to 150 m, *medium* size birds up to 250 m and *large* birds up to 300 m.

The constructed prototype composed of eight *Detection Modules* and one *Decision-Making System* was installed at the wind turbine in northern Poland. Two kinds of tests where applied. First, the system was validated using a bird-like GPS equipped drone of wingspan 2.0 m. The averaged drone localization uncertainty error (2.85 m) was below theoretical quantization error (3.85 m) during the flight at 143.3 m from turbine.

Secondly, the results of ornithologists’ long-term observations were compared with the system records. During an 67.5 hour observation, the ornithologists identified 105 of *small*, *medium* and *large* birds. In this period the system detects 96 birds. All 9 missed object where observed at the larger distances (>150 m). More importantly, within the 100 m range, all birds observed by ornithologists were also detected by the system. At a distance between 100 m and 200 m only one medium size bird was not detected by the system. Furthermore, in one case of 98 birds, the system misclassified a bird into a lower class than the ornithologist. The test proved the required performance quality of the developed detection, localization and classification algorithms.

The system configuration and customization abilities allow its adjustment to desired requirements. The detection range of the presented solution was designed to cover the requested 300 m observation zone for *large* birds such as Red Kite. However, some authorities have recently introduced an even more restricting obligation with a detection range of up to 500 m [16]. Nevertheless, through the applied distributed computing paradigm and modular construction, the system could be easily re-configured to cover even more challenging observation zone.

Although the system was verified by ornithologists at random dates, different times of the day and weather conditions, there is still a need for a more systematized system’s performance analysis under various overcast conditions. Also, the reliability of bird flock detection should be evaluated. These are the areas of our future work.

Future development may concern a tracking algorithm to anticipate bird flight paths to decrease unnecessary turbine stopping. Furthermore, implementation of the Kalman filter, Probability Hypothesis Density (PHD) filter or Multiple Hypothesis Tracking (MHT) can improve the localization measurement accuracy and thus bird classification. Due to the fuzziness of bird size classes, the classifier may be enhanced by applying a fuzzy logic approach.

## Figures and Tables

**Table 1:** Comparison of vision techniques for bird detection by DT Bird, SafeWind, Identyflight, BirdVision, Airelectronics.

Method	DT Bird	SafeWind	Identyflight	BirdVision	Airelectronics
Detection method	Monoscopic	Monoscopic	Stereoscopic	Monoscopic	Monoscopic
Distance estimation	No	No	Yes	No	Yes
Localization	No	No	Stereo-vision-based	No	No
Maximum detection range	650 m	-	1500 m	300 m	600 m
Target classification	No	No	Golden & Bald Eagles, Red Kite	No	No
Installation	Wind turbine	Wind turbine	Separate tower	Wind turbine	Wind turbine
Collision prevention	Audio, Turbine stop	Audio, Turbine Stop	Turbine stop	Turbine stop	Audio, Turbine stop

**Table 2:** Comparison of CNN architectures used for bird identification. BN—Batch Normalization; SC—Skip Connection; FP—False Positives.

Paper	Database	Identification Algorithm	Image Size [px × px × Channel]	Pooling Window	Activation Function	Identification Accuracy
[47]	[42]	CNN	28 × 28 × 3	max 2 × 2	ReLU	80–90%, 0.2 FP
[45]	[45]	CNN	256 × 256 × 1	-	softmax	90–98%, 0.2 FP
[48]	[49]	CNN (BN, SC)	112 × 112 × 1	max 2 × 2	ReLU, softmax	90–99%
[4]	[4]	CNN (SC)	128 × 128 × 1; 96 × 96 × 1; 64 × 64 × 1; 32 × 32 × 1	-	-	70–90%

**Table 3:** Functional and nonfunctional requirements and particular constrains.

		General Requirements	Itemized Requirements	Particular Constrains
Environmental Authorities			Rare bird species	Very high effectiveness
		Protection of the birds	Big birds	High effectiveness
			Medium/Small birds	Medium effectiveness
			During daylight	>100 lux
		Collision avoidance	Turbine stopping	Compulsory for rare and big birds
			Deterrence	Optional for further distances
		Validation data	Photo and video from events	High resolution allowing bird identification, data storage for 1 year
Wind farm developers	Functional	Bird localization	Distance estimation	90% accuracy
		Bird classification/	Size	Small/Medium/Large
		identification	Species	Local rare species
				High classification reliability
		Collision avoidance	Turbine stopping	Minimization of turbine-off time
			Deterrence method	Audio/Strobo
		User interface	Easy access	Web/mobile application
	Nonfunctional	Installation	Non-invasive installation	On turbine using stainless steel climbs
		System lifetime	As long as possible	Minimum five years
		System verification	Data from the events	High-resolution photos
				High-resolution color smooth video
			Collision monitoring	At least HD resolution smooth Video
		System accessibility	Web App	Chrome, Mozilla, Safari
			Mobile App	Android, IOS
		Data handling	Storage	At least 2 years
			Reports	Selective allowing the choice of only interesting data up to a year back
Manufacturer	Functional	Installation	Plug and Play Solution	Module construction, easy replacement
			Compatibility with existing systems	Turbine stop using PLC or SCADA trough ModBus.
		Maintenance	Remote software upgrade	IoT.
			In situ auto calibration	Daily.
	Nonfunctional	High reliability	Small number of FP	Annual average number of FP less than 10% of all detections.
		Remote connection	Daily status check	Fast and secure.
		Customizability	Adjustment of system parameters such as detection range and size classification criteria	To bird species nesting nearby, local law and regulations, specific ornithology’s recommendations, turbines features.

**Table 4:** Optical parameters of selected vision sensors.

Parameter	Unit	C1 (IMX219)	C2 (IMX447)	C3 (AR1335)	C4 (AR1820HS)
$VSR_{h}$	px	3280	4056	4208	4912
$VSR_{v}$	px	2464	3040	3120	3684
$VSS_{h}$	mm	3.680	6.287	6.300	7.660
$VSS_{v}$	mm	2.760	4.712	5.700	4.560
$VSR_{h}$/$VSS_{h}$	px/mm	891.30	645.14	667.93	641.25
$VSR_{v}$/$VSS_{v}$	px/mm	892.75	645.16	547.36	807.95

**Table 5:** Impact of the lens on the camera detection capabilities. The parameters, which fulfill the requirements are in bold. The selected options are underlined.

*f* [mm]	C1		C2		C3		C4	
	${\mathrm{FoV}}_{v/h}$	$p_{W/H}$	${\mathrm{FoV}}_{v/h}$	$p_{W/H}$	${\mathrm{FoV}}_{v/h}$	$p_{W/H}$	${\mathrm{FoV}}_{v/h}$	$p_{W/H}$
	**[${}^{\circ }$] × [${}^{\circ }$]**	**[px]** × **[px]**	**[${}^{\circ }$] × [${}^{\circ }$]**	**[px]** × **[px]**	**[${}^{\circ }$] × [${}^{\circ }$]**	**[px]** × **[px]**	**[${}^{\circ }$] × [${}^{\circ }$]**	**[px]** × **[px]**
3	**63.0 × 49.4 **	**13.0 × 2.0**	92.7 × 76.3	10.0 × 1.0	92.8 × 87.1	10.0 × 1.0	103.9 × 74.5	9.0 × 2.0
4	49.4 × 38.1	18.0 × 2.0	**76.3 × 61.0**	**13.0 × 2.0**	76.4 × 70.9	13.0 × 1.0	**87.5 × 59.4**	**13.0 × 2.0**
6	34.1 × 42.9	26.0 × 4.0	55.3 × 42.9	19.0 × 3.0	55.4 × 50.8	20.0 × 2.0	**65.1 × 41.6**	**19.0 × 3.0**
8	25.9 × 32.8	35.0 × 5.0	42.9 × 32.8	26.0 × 3.0	43.0 × 39.2	27.0 × 3.0	51.2 × 31.8	26.0 × 4.0
12	17.4 × 22.2	53.0 × 7.0	29.4 × 22.2	39.0 × 5.0	29.4 × 26.7	40.0 × 4.0	35.4 × 21.5	38.0 × 6.0
16	13.1 × 16.8	71.0 × 10.0	22.2 × 16.8	52.0 × 7.0	22.3 × 20.2	53.0 × 6.0	26.9 × 16.2	51.0 × 9.0

**Table 6:** Test results of CNN performance evaluation, where the bolded row highlights the selected configuration; the values in red highlight the best values for a given parameter.

CNN Parameters			*FF Time* [ms]	*Precision*	*Recall*	*F*1	*Specificity*	*Accuracy*
$L_{C1}$	$L_{C2}$	$L_{\mathrm{FC}}$						
32	32	32	0.80	0.987	0.989	0.988	0.987	0.988
32	32	64	0.97	0.990	0.989	0.989	0.990	0.989
**32**	**32**	**128**	**1.09**	**0.996**	**0.989**	**0.993**	**0.996**	**0.993**
32	32	256	1.59	0.995	0.989	0.992	0.995	0.992
32	64	32	1.28	0.995	0.989	0.992	0.995	0.992
32	64	64	1.42	0.998	0.988	0.993	0.998	0.993
32	64	128	1.93	0.995	0.989	0.992	0.995	0.992
32	64	256	2.85	0.998	0.989	0.994	0.999	0.994
64	32	32	1.54	0.979	0.989	0.984	0.979	0.984
64	32	64	1.65	0.961	0.989	0.975	0.960	0.975
64	32	128	1.84	0.997	0.989	0.993	0.997	0.993
64	32	256	2.31	0.987	0.989	0.988	0.987	0.988
64	64	32	2.33	0.997	0.989	0.993	0.997	0.993
64	64	64	2.51	0.994	0.989	0.992	0.994	0.992
64	64	128	3.32	0.987	0.989	0.988	0.987	0.988
64	64	256	3.84	0.998	0.989	0.994	0.998	0.994
min			0.80	0.961	0.989	0.975	0.960	0.975
max			3.84	0.998	0.989	0.994	0.999	0.994

**Table 7:** Classification boundaries of small, medium and large birds.

Class	Detection Range [m]	Wingspan [m]	Height [m]	Size [$m^{2}$]	Example Bird
Uncategorized	-	<0.68	<0.32	<0.11	Feral Pigeon
					House Sparrow
Small	10–183	0.68–1.25	0.32–0.39	0.11–0.24	Common kestrel
					Peregrine Falcon
Medium	10–312	1.26–1.50	0.40–0.55	0.25–0.41	Steppe Buzzard
					Marsh Harrier
Large	10–392	>1.50	>0.55	>0.41	Red Kite
					White stork

**Table 8:** Results of distance and size measurements at different distances for silhouettes simulating *small*, *medium* and *large* birds.

Parameter	Unit	Reference	Reference Distance $D_{b_{\mathrm{ref}}}$ [m]					
		Value	50.00	100.00	150.00	200.00	250.00	300.00
$y_{diff}$	px	-	54	27	19	-	-	-
$D_{b}$	m		50.35	100.69	143.09	-	-	-
$\Delta D_{b_{ref}}$	m		0.43	1.86	3.77	-	-	-
$D_{b_{ref}}-D_{b}$	m		0.35	0.69	6.91	-	-	-
$P_{W}$	m	0.80	0.84	0.83	0.75	-	-	-
$P_{H}$	m	0.30	0.34	0.44	0.32	-	-	-
$O_{s}$	m${}^{2}$	0.12	0.16	0.18	0.13	-	-	-
$O_{approx}$	m${}^{2}$		0.14	0.19	0.12	-	-	-
$y_{diff}$	px	-	55	28	18	13	10	-
$D_{b}$	m		49.43	97.09	151.04	209.13	271.87	-
$\Delta D_{b_{ref}}$	m		0.43	2.17	4.20	8.04	13.59	
$D_{b_{ref}}-D_{b}$	m		0.57	2.91	1.04	9.13	21.87	-
$P_{W}$	m	1.20	1.18	1.16	1.24	1.09	1.22	-
$P_{H}$	m	0.40	0.41	0.44	0.51	0.39	0.41	-
$O_{s}$	m${}^{2}$	0.24	0.24	0.24	0.33	0.23	0.23	-
$O_{approx}$	m${}^{2}$		0.23	0.32	0.32	0.21	0.25	-
$y_{diff}$	px	-	54	26	19	13	10	9
$D_{b}$	m		50.35	104.56	143.09	209.13	271.87	302.07
$\Delta D_{b_{ref}}$	m		0.40	2.01	3.77	8.04	13.59	16.78
$D_{b_{ref}}-D_{b}$	m		0.35	4.56	6.91	9.13	21.87	2.07
$P_{W}$	m	1.50	1.42	1.56	1.50	1.49	1.53	1.36
$P_{H}$	m	0.50	0.51	0.51	0.48	0.47	0.51	0.45
$O_{s}$	m${}^{2}$	0.38	0.42	0.39	0.36	0.38	0.40	0.38
$O_{approx}$	m${}^{2}$		0.36	0.40	0.36	0.35	0.39	0.31

**Table 9:** Summary of drone test results.

	$\bar{D}$ [m]	$\sigma_{D}$ [m]	$\bar{H}$ [m]	$\sigma_{H}$ [m]	$D_{b_{\min}}$ [m]	$D_{b_{\max}}$ [m]
Module A	146.1	7.7	104.1	4.4	148.7	178.4
Module B	146.2	8.4	106.7	5.5	140.9	178.4
Drone	143.3	1.2	102.9	0.6	-	-

**Table 10:** Comparison of ornithologists’ observations and system’s identification and classification of selected bird species.

Species Name	Wingspan [m]	Identification Rate (Sys/Ornithologist)			System Classification
[Eng]/[Lat]	[63]	<100 m	<100 m–200 m>	>200 m	Small/Medium/Large
Wood pigeon/	0.67–0.77	5/5	5/5	0/1	8/2/0
Columba palumbus					
Common buzzard/	1.10–1.30	6/6	4/4	-	5/3/2
Buteo					
Raven/	1.15–1.30	24/24	18/19	22/28	14/22/30
Corvus corax					
Marsh harrier/	1.15–1.40	2/2	1/1	1/1	2/1/1
Circus aeruginosus					
Herring gull/	1.23–1.48	2/2	1/1	-	1/1/1
Larus argentatus					
Red Kite/	1.40–1.65	1/1	-	1/1	0/2/0
Milvus					
Crane/	1.80–2.22	-	2/2	1/2	0/1/2
Grus					

## Data Availability

Presented data accessible for authorised staff accordingly to local regulation.
